# Comprehensive analysis of clustering algorithms: exploring limitations and innovative solutions

**DOI:** 10.7717/peerj-cs.2286

**Published:** 2024-08-29

**Authors:** Aasim Ayaz Wani

**Affiliations:** School of Engineering, Cornell University, Ithaca, New York, United States

**Keywords:** Clustering algorithms, Unsupervised learning, Scalability and efficiency, Centroid-based clustering, Hierarchical clustering, Density-based clustering, Distribution-based clustering, Clustering challenges and solutions

## Abstract

This survey rigorously explores contemporary clustering algorithms within the machine learning paradigm, focusing on five primary methodologies: centroid-based, hierarchical, density-based, distribution-based, and graph-based clustering. Through the lens of recent innovations such as deep embedded clustering and spectral clustering, we analyze the strengths, limitations, and the breadth of application domains—ranging from bioinformatics to social network analysis. Notably, the survey introduces novel contributions by integrating clustering techniques with dimensionality reduction and proposing advanced ensemble methods to enhance stability and accuracy across varied data structures. This work uniquely synthesizes the latest advancements and offers new perspectives on overcoming traditional challenges like scalability and noise sensitivity, thus providing a comprehensive roadmap for future research and practical applications in data-intensive environments.

## Introduction

Clustering algorithms constitute a fundamental component of unsupervised machine learning, facilitating the discovery of hidden patterns and structures within unlabeled datasets. These algorithms partition data points into distinct groups or clusters based on their inherent similarities, ensuring that points within a cluster are more similar to each other than to those in other clusters. These techniques are critical across diverse fields such as bioinformatics, image segmentation, anomaly detection, and customer segmentation (Lan et al., 2018; Sofi & Wani, 2021; Feng et al., 2023). These applications underscore the significant role of clustering in extracting valuable insights from the vast amounts of data generated daily (Jun, Yoo & Choi, 2018; Xu & Tian, 2015). But, despite their widespread application, clustering algorithms often face significant challenges when dealing with high-dimensional, noisy, and large-scale data.

While previous surveys have provided valuable overviews of various clustering algorithms, the rapid advancements in the field necessitate an updated and comprehensive analysis of the latest techniques, their limitations, and innovative solutions (Fahad et al., 2014; Xu & Tian, 2015). This survey article aims to bridge this gap by providing an in-depth examination of both classical and state-of-the-art clustering algorithms, with a particular focus on their methodologies, strengths, and weaknesses. Moreover, we identify and discuss key challenges faced by clustering algorithms, such as the curse of dimensionality, initialization sensitivity, and scalability issues, and propose advanced solutions to overcome these obstacles. The main objectives and contributions of this survey are as follows:

- Provide a comprehensive and up-to-date analysis of various clustering techniques, including centroid, hierarchical, density, distribution, autoencoders and graph-based clustering methods.
- Discuss the methodologies, strengths, and limitations of each category of clustering algorithms, along with their practical applications across multiple domains.
- Identify key challenges and limitations of existing clustering algorithms.
- Propose and analyze advanced solutions to address these challenges, including dimensionality reduction techniques, ensemble clustering, and other state-of-the-art approaches.
- Highlight the importance of integrating clustering with other machine learning paradigms and emphasize the need for robust validation metrics to assess clustering outcomes effectively.

This article aims to bridge the gap between classical clustering methods and contemporary advancements by providing a comprehensive analysis of both traditional and state-of-the-art clustering algorithms. Our goal is to stimulate further research and development of clustering algorithms that are more efficient, robust, and adaptable to the complexities of real-world data. By addressing these issues and highlighting the importance of integrating clustering with other machine learning paradigms, we aim to contribute valuable insights and foster advancements in the field. This survey serves as a resource for researchers and practitioners, offering guidance on the selection and application of clustering techniques tailored to specific data characteristics and analytical needs.

The remainder of this article is organized as follows: “Categorization of Clustering Algorithms” details various clustering methods discussing their methodologies and applications. “Practical Challenges of Existing Clustering Methods” explores the limitations and challenges faced by current clustering algorithms in various application scenarios. “Solutions for Overcoming Clustering Limitations” proposes innovative solutions and advanced methodologies to address these challenges. Finally, “Conclusions and Future Work” summarizes the findings of this survey and discusses potential future research directions in the field of clustering algorithms.

### Survey/search methodology

To ensure comprehensive and unbiased coverage of the literature, we employed a systematic and rigorous search methodology. We utilized multiple reputable search engines and academic databases, including Google Scholar, PubMed and IEEE Xplore chosen for their extensive coverage of computer science and data analysis research. Our search used a combination of terms such as “clustering algorithms”, “centroid-based clustering”, “K-means clustering”, “hierarchical clustering,” “density-based clustering”, “distribution-based clustering”, “Gaussian Mixture Models”, “graph-based clustering”, “clustering in high-dimensional data”, “clustering performance evaluation” and “clustering challenges and solutions”. Boolean operators (AND, OR) refined the queries to include studies directly addressing our research questions. Inclusion criteria were articles that focused on clustering algorithms and their applications, published within the last 15 years, peer-reviewed, and written in English. The resulting articles found were then sorted based number of citations. Exclusion criteria involved studies not centered on clustering algorithms, older than 15 years unless seminal, non-peer-reviewed, or in languages other than English. The search process began with a broad search using the specified terms. Titles and abstracts of the retrieved articles were screened for relevance, and those not meeting the criteria were discarded. Full texts of the remaining articles were reviewed to ensure they met all inclusion criteria. References of selected articles were also checked to identify additional relevant studies.

## Categorization of clustering algorithms

### Connectivity models: hierarchical clustering

Connectivity-based models, leverage structure within datasets to identify tree-like relationships that illustrate the hierarchical relationship between clusters. Hierarchical clustering has two primary approaches: agglomerative (bottom-up) and divisive (top-down). In the hierarchical agglomerative clustering (HAC), the algorithm starts by treating each data point as its own cluster and iteratively merges the most similar pairs of clusters into successively larger clusters, while the divisive approach takes the opposite strategy.

Clusters are merged by assessing the similarity of their centroids based on proximity in feature space, merging those with the highest similarity according to the chosen linkage criterion. The similarity between data points is quantified using distance measures such as Euclidean, Manhattan similarity *etc*. (Jain & Dubes, 1988; Hastie et al., 2009). The merging process employs various linkage criteria to recalculate distances between clusters. If $x$ and $y$ are two data points in an $n$-dimensional space, Mathematically:

(1) $${\mathrm{Distance}} = \left\{ {\matrix{ {\sqrt {\sum\nolimits_{i = 1}^n {{{({x_i} - {y_i})}^2}} } } & {({\mathrm{Euclidean}}\;{\mathrm{Distance}})} \cr {\sum\nolimits_{i = 1}^n | {x_i} - {y_i}|} & {({\mathrm{Manhattan}}\;{\mathrm{Distance}})}. \cr } } \right. $$

In HAC, the linkage criterion is crucial as it determines how distances between clusters are calculated, which in turn affects cluster assignments and the overall outcome of the clustering process. Different linkage criteria influence the shape and size of clusters, each having unique objective functions and stopping criteria that significantly impact the resulting dendrogram shapes (Bishop, 2006). There are primarily five types of linkage criteria: single, complete, average, centroid linkage, and Ward’s method (Yim & Ramdeen, 2015; Schubert, 2021).

- **Ward’s linkage:** This method creates clusters of roughly equal sizes by minimizing the increase in total within-cluster variance at each step of merging. It tends to produce more balanced and high density clusters with nearly uniform density.
- **Complete linkage:** It forms clusters based on the maximum distance between observations in different clusters. This criterion leads to tighter, more compact clusters and tends to delay the merging of geographically distant clusters until necessary.
- **Average linkage:** This method uses the average distance between all pairs of observations in different clusters. It provides a balance between the characteristics of single and complete linkage, employing a moderate merging criterion.
- **Single linkage:** Based on the minimum distance between any members of two clusters, this method can lead to a “chaining” effect. Clusters grow by merging with other clusters that have even just one close member, often resulting in elongated, chain-like clusters.

(2) $$ d(S,T) = \left\{ {\matrix{ {\min (||x - y||:x \in S,y \in T)}  & {{\mathrm{for}}\;{\mathrm{single}}\;{\mathrm{linkage}}}\quad\;  \cr {\max (||x - y||:x \in S,y \in T)}  & {{\mathrm{for}}\;{\mathrm{complete}}\;{\mathrm{linkage}}}  \cr {{1 \over {|S||T|}}\sum\nolimits_{x \in S,y \in T} {||} x - y||}\quad\quad  & {{\mathrm{for}}\;{\mathrm{average}}\;{\mathrm{linkage}}}\;\;  \cr {{{|S||T|} \over {|S| + |T|}}||{\mu _S} - {\mu _T}||^{2}}\quad\quad\quad\quad  & {{\mathrm{for}}\;{\mathrm{Ward}'} {\mathrm{s}}\;{\mathrm{method}}}\;  \cr } } \right.$$

(3) $$d(S \cup T,U) = \min (d(S,U),d(T,U))$$

where *S*, *T*, and *U* are clusters and $d$ represents the distance between them defined by the criterion.

HAC typically has a high time complexity of $O({n^3})$ in its basic form (Kaufman & Rousseeuw, 2009). The algorithm builds a hierarchy of clusters visualised by dendogram, by iteratively merging the nearest clusters until a single cluster or a stopping criterion is met, requiring frequent distance updates. Optimized data structures, like priority queues, can reduce this complexity to $O({n^2}\log n)$ (Dhulipala et al., 2023).

Stopping criteria for HAC include reaching set number of clusters, exceeding dissimilarity threshold, or limiting dendrogram height to maintain distinct clusters. Algorithms like CURE and BIRCH improve on traditional HAC by minimizing within-cluster variance and accommodating non-spherical shapes and varying densities (Zhang, Ramakrishnan & Livny, 1996; Guha, Rastogi & Shim, 1998). Divisive algorithms like DIANA and MONA offer further adaptability to different data structures (Kaufman & Rousseeuw, 2009). Improvements to HAC address scalability, cluster overlap, and high-dimensionality challenges (Ding & He, 2004).

### Centroid models: k-means

Centroid-based clustering, a prominent class of partitioning methods, organizes data points into clusters based on their proximity to representative centroids. These centroids characterize the core features of each cluster and are typically computed as the mean or median of the points within a cluster. Objective Function: The k-means algorithm aims to minimize the within-cluster sum of squares (WCSS), which is the sum of squared distances between data points and their respective cluster centroids, which quantifies the variance within each cluster resulting in clusters with no overlap, spherical shaped, with uniform density of points around the cluster centroids. Mathematically this algorithm can be defined as:

(4) $$J = \sum\limits_{i = 1}^k {\sum\limits_{{\bf{x}} \in {S_i}} | } {\bf{x}} - {{{\mu }}_i}|^{2}\quad {\mathrm{where}}\quad {{{\mu }}_i} = {1 \over {|{S_i}|}}\sum\limits_{{\bf{x}} \in {S_i}} {\bf{x}} \quad {\mathrm{and}}\quad i = \arg {\min _j}|{\bf{x}} - {{{\mu }}_j}|^{2}.$$

**Figure 1:**
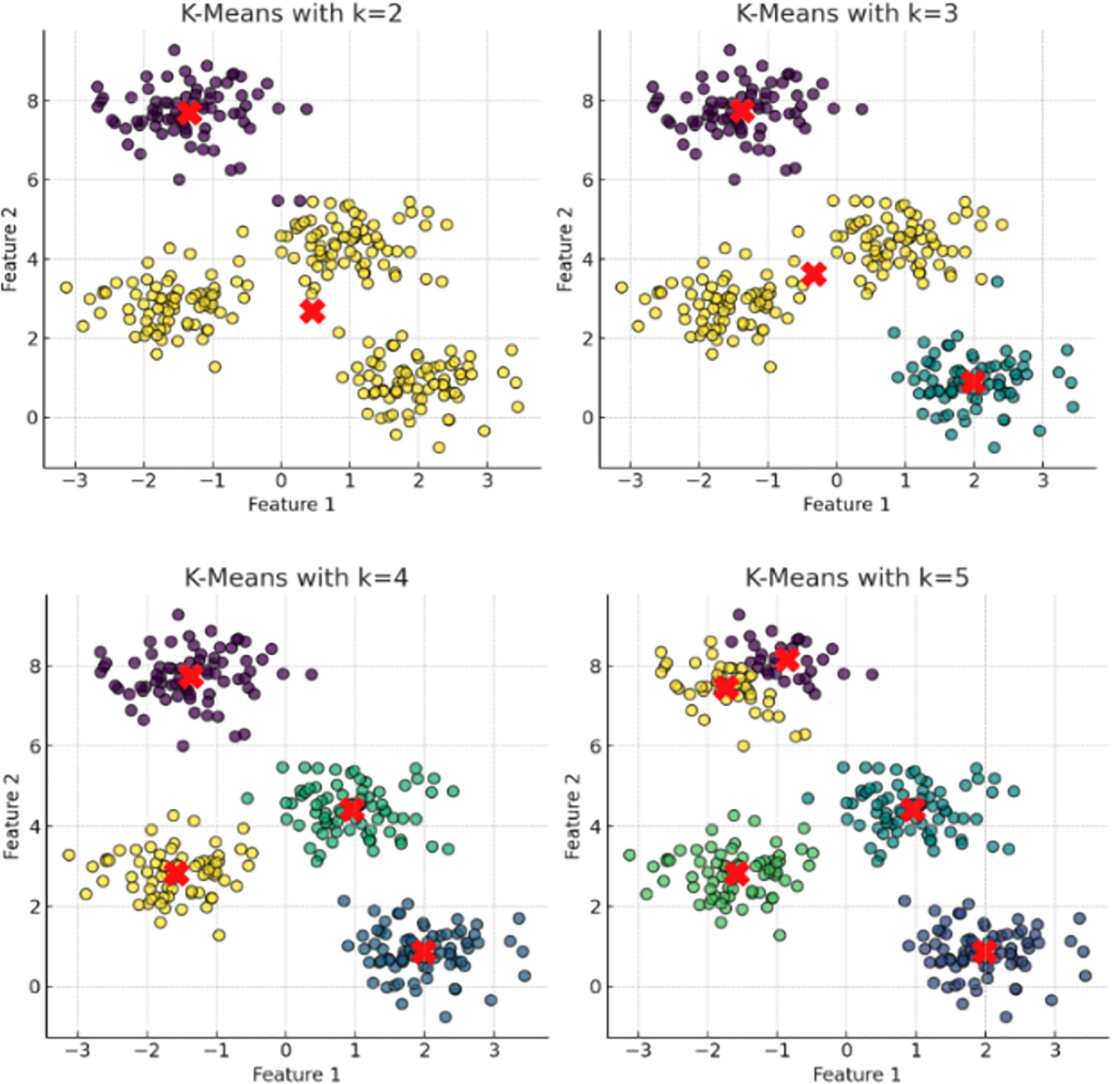
The plot has been generated using simulated data from sklearn.datasets ‘make blobs’. These plots depict the results of applying the K-means clustering algorithm with incremental cluster counts (k = 2, 3, 4, 5) to a multidimensional dataset. Each panel represents the clusters identified by the algorithm with centroids marked by red crosses. The progression from k = 2 to k = 5 demonstrates the algorithm’s behavior in partitioning the data into increasingly specific groups based on the Euclidean distances between data points. This visualization serves to underscore the potential for over-segmentation inherent in K-means when increasing k without employing a rigorous method to determine the optimal number of clusters, such as the elbow method or silhouette scores. This sequence of clustering highlights the critical balance between capturing genuine data structure and avoiding the imposition of artificial divisions within the dataset.

K-means clustering relies on accurate distance measurements between data points and cluster centers, influenced by the choice of distance metric and centroid initialization. Selecting a suitable distance metric aligns the clustering method with data characteristics. Euclidean distance, the default for k-means, minimizes intra-cluster variance, forming spherical clusters. Manhattan distance creates diamond-shaped or hyper-rectangular clusters, suitable for certain datasets (Hastie et al., 2009). Aligning the metric with data nature and distribution is crucial. For instance, Fig. 1 shows k-means misidentifying two concentric circles as separate clusters with uniform density and spherical shape.

K-means require a predetermined number of clusters ( $k$), significantly influencing the dataset’s final partitioning. Incorrect estimation of $k$ can lead to sub-optimal or misleading cluster assignments (Liu, Lu & Zhang, 2020). The elbow method is a common strategy for determining an appropriate $k$. This involves calculating the WCSS for a range of $k$ values and plotting WCSS against $k$. The optimal $k$ is identified at the elbow point of the curve, where the rate of WCSS decrease levels off, balancing intra-cluster variance and avoiding overfitting (Fig. 1).

Despite k-means clustering’s guaranteed convergence, it often falls into local minima due to its reliance on random centroid initialization, classifying it as a greedy algorithm. This can result in suboptimal clustering solutions or increased convergence times. A common strategy to address this issue is to run k-means multiple times with different initializations and select the solution with the lowest WCSS, which helps in finding a better global optimum. Several advanced techniques have been developed to mitigate the local minima problem in k-means clustering. These include:

- **Repeated random initializations** Running k-means multiple times with different random starting points and choosing the best result (Fränti & Sieranoja, 2019).
- **k-means++** method strategically initializes centroids to ensure better initial separation. This approach improves convergence speed and reduces the likelihood of poor local optima (Jain, 2010; Steinley, 2006; Arthur, 2007)

The k-means algorithm has a time complexity of $O(kndi)$, where $k$ is number of clusters, $n$ is number of data points, $d$ is the dimensionality, and $i$ is number of iterations until convergence. K-means iteratively assigns data points to the nearest centroid and updates centroids based on new cluster memberships.

Refined initialization methods, such as those by Bradley & Fayyad (1998) estimate distribution modes from small sample clusters, enhancing scalability for large datasets. Adaptations for mixed data types include modified cost functions, kernel functions for categorical data, and various dissimilarity measures (Jiacai & Ruijun, 2010; Couto, 2005; Bai et al., 2012). Further research includes robust centroid estimation techniques like trimmed K-means and M-estimators to mitigate outlier influence (Cuesta-Albertos, Gordaliza & Matrán, 1997; García-Escudero et al., 2008). Density-aware approaches, such as DBCV and DENCLUE, use density information to identify clusters of varying shapes and densities, addressing the spherical cluster assumption (Khan & Ahmad, 2013; Campello et al., 2015). Ensemble techniques like bagging and boosting combine multiple clustering models to enhance robustness and stability (Strehl & Ghosh, 2002; Fern & Brodley, 2003).

### Density-based clustering: DBSCAN

**Figure 2:**
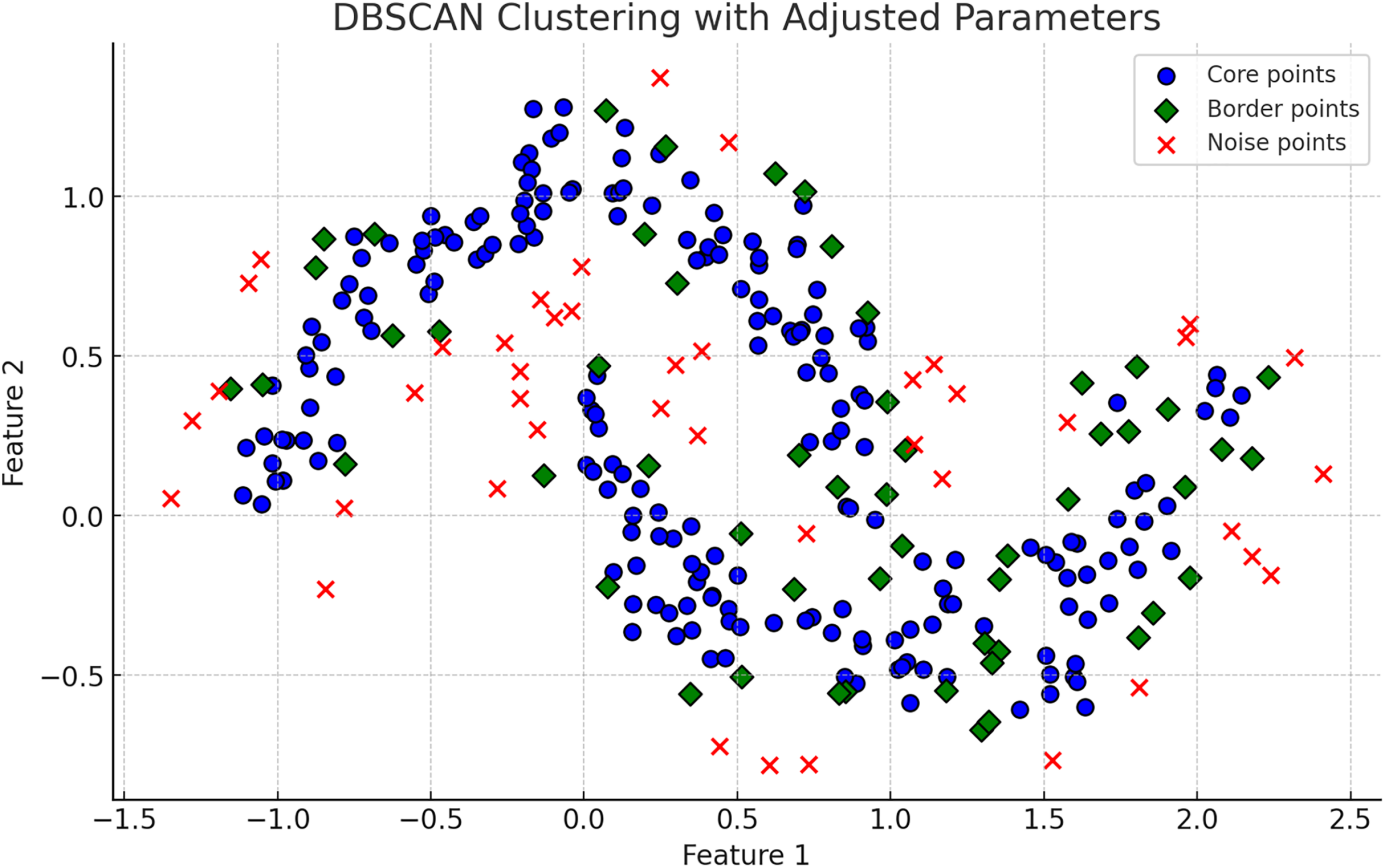
The plot has been generated using simulated data from sklearn.datasets ‘make moons’ using the two-dimensional scatter plots. Operational mechanics of DBSCAN Clustering: Illustrates the classification of points into core, border, and noise categories within DBSCAN, showing the algorithm’s robustness to noise and its ability to form arbitrarily shaped clusters.

**Figure 3:**
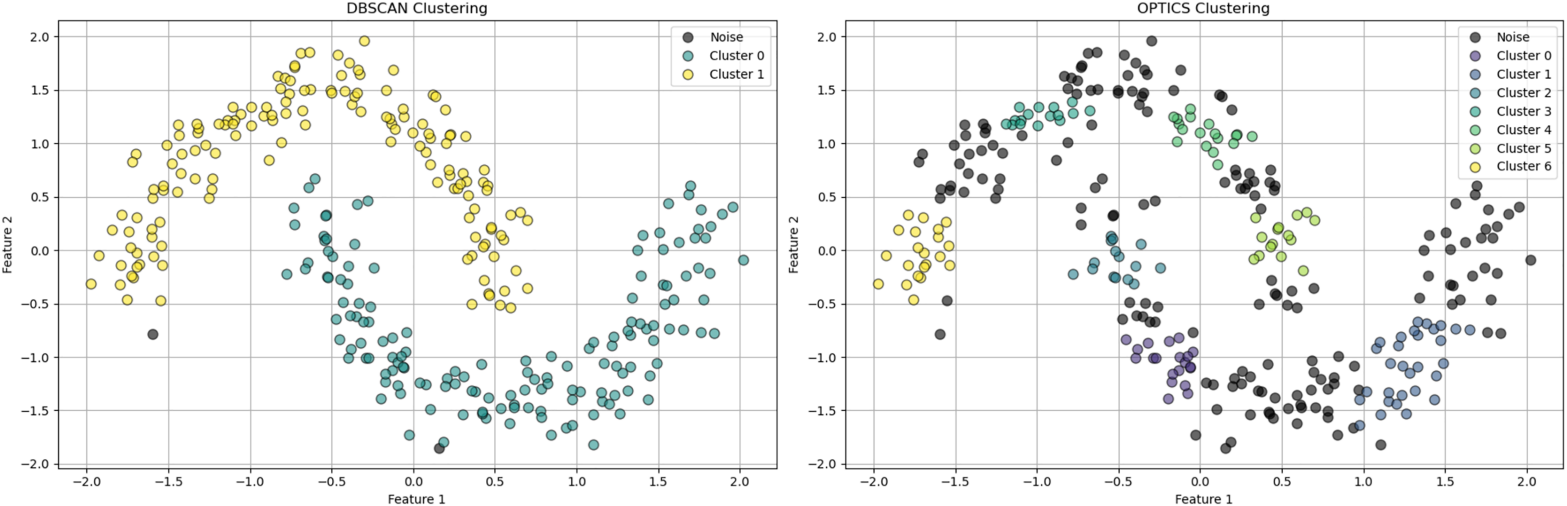
The plot has been generated using simulated data from sklearn.datasets ‘make moons’ using the two-dimensional scatter plots. The plot is highlighting the classification of data points in cluster Analysis: Depicts core, border, and noise classifications typical in density-based clustering algorithms.

Density-based clustering algorithms are integral in machine learning due to their ability to identify clusters of arbitrary shapes and effectively handling noise and outliers. The core concepts of DBSCAN revolve around two parameters: Epsilon ( $\varepsilon$), a distance threshold determining the neighborhood around a data point, and minimum points ( $MinPts$), the minimum number of points required within an $\varepsilon$-radius to consider the region dense. DBSCAN (Density-Based Spatial Clustering of Applications with Noise), introduced by Ester et al. (1996) is a foundational algorithm forming clusters based on data point density. OPTICS (Ordering Points To Identify the Clustering Structure) builds on DBSCAN by removing the need for a global reachability distance, using a reachability plot to adapt to local density variations, enhancing utility in datasets with varying density clusters (Ankerst et al., 1999). HDBSCAN extends DBSCAN by using a hierarchical approach that does not require $\varepsilon$ specification, determining the best clustering solution based on cluster stability over different scales, offering improved flexibility over traditional DBSCAN (Campello, Moulavi & Sander, 2013). In Fig. 2, DBSCAN demonstrates its effectiveness in distinguishing noise from significant clusters by classifying points as core, border, or noise points. Figure 3, highlights difference between DBSCAN and Optics.

- Core Points: Points with enough neighbors within the $\varepsilon$-distance to form a dense region, mathematically defined as:
(5) $$|{N_\varepsilon }(p)| \ge {\mathrm{MinPts}}$$
- Border Points: Points not classified as core points but reachable from a core point, mathematically defined as:
(6) $$|{N_\varepsilon }(p)|{\mathrm{ \;\lt\; MinPts}}\quad {\mathrm{and}}\quad \exists q \in {N_\varepsilon }(p):|{N_\varepsilon }(q)| \ge {\mathrm{MinPts}}$$
- Noise Points: Points classified as neither core nor border points, mathematically defined as:
(7) $$|{N_\varepsilon }(p)|{\mathrm{ \;\lt\; MinPts}}\quad {\mathrm{and}}\quad \forall q \in {N_\varepsilon }(p),|{N_\varepsilon }(q)|  \;< \; {\mathrm{MinPts}}$$

This feature is particularly valuable in noisy datasets, where methods like K-means might mistakenly include noise points within clusters. Unlike centroid-based or distribution-based clustering, DBSCAN does not require assumptions about the underlying cluster distribution, making it ideal for applications with unknown or evolving data distributions, such as geographic data (Miraftabzadeh et al., 2023). Where:

-
${N_\varepsilon }(p)$ is the $\varepsilon$-neighborhood of $p$, containing all points within $\varepsilon$ distance from $p$.
-
$\varepsilon$ is the maximum radius of the neighborhood around each data point.
-
${\mathrm{MinPts}}$ is the minimum number of points required to form a dense region.
-
$q$ is a core point within $p$’s $\varepsilon$-neighborhood.

The time complexity of density-based clustering algorithms, such as DBSCAN, OPTICS, and HDBSCAN, ranges from $O(n\log n)$ to $O({n^2})$, depending on the number of data points $n$ and the use of spatial indexing. DBSCAN and OPTICS achieve an average complexity of $O(n\log n)$ using efficient data structures like kd-trees or R-trees for neighborhood queries (Schubert et al., 2017). Without such optimizations, the complexity can escalate to $O({n^2})$ due to pairwise distance computations. HDBSCAN follows a similar pattern, with hierarchy construction in $O(n\log n)$ and subsequent cluster extraction in $O(n)$. The complexity of the DENCLUE algorithm varies with implementation and can fall within the same range, depending on the density estimation techniques used (Khan & Ahmad, 2013; Campello et al., 2015).

For density-based clustering algorithms like DBSCAN, the stopping criteria are defined by the algorithm’s parameters, such as $\varepsilon$ and MinPts, conclusion happens when all points have been evaluated within their local density context (Al-mamory & Kamil, 2019). This non-iterative approach highlights DBSCAN’s efficiency in handling data with varying densities and shapes, compared to k-Means, which may struggle with non-uniform density and is biased towards circular clusters (Chowdhury, Helian & de Amorim, 2023).

### Distribution model based: gaussian mixture models

Gaussian mixture models (GMM) assume data points are generated from a mixture of Gaussian distributions, each characterized by its mean ( $\mu$) and covariance ( $\Sigma$). This approach allows GMMs to adapt to complex data structures with varying shapes and is particularly effective for modeling multi-modal distributions where simpler models, like k-means, might fail (Wang & Jiang, 2021). Parameters of GMMs are typically estimated using the Expectation-Maximization (EM) algorithm, which iteratively adjusts parameters to maximize the likelihood of the data given the model.

The performance and complexity of GMMs depends on the initial parameter estimates ( $\mu$, $\Sigma$, and mixture weights $\pi$). Poor initial guesses can lead to slow convergence and suboptimal solutions. The iterative nature of the EM algorithm involves repeated updates, making the process computationally intensive, especially with a large number of clusters or high-dimensional datasets. The number of parameters grows quadratically with dimensionality due to the covariance matrices, leading to potential overfitting, due to curse of dimensionality (Diallo, Morin & Lu, 2016).

The EM algorithm converges based on a stopping criterion, typically the change in log-likelihood between successive iterations. The algorithm halts when this change falls below a predefined threshold, indicating minimal improvement. The time complexity of the GMM is influenced by the number of iterations (*I*), mixture components (*K*), data points (*N*), and data dimensionality (*D*), generally resulting in $O(I \cdot K \cdot N \cdot {D^2})$ complexity due to the quadratic dependence on *D* (Bishop, 2006).

The EM algorithm alternates between assigning data points to clusters (Expectation step) and updating distribution parameters (Maximization step) to optimize the model’s fit (Redner & Walker, 1984). However, convergence issues and the risk of local optima necessitate careful initialization strategies and algorithmic refinements. Effective initialization techniques, such as k-means++ for initial cluster centers, significantly improve the EM algorithm’s robustness and accuracy (Arthur, 2007). Additionally, annealing methods and optimization techniques like variational inference can help mitigate the risk of poor local optima, ensuring a more reliable clustering outcome (Blei, Kucukelbir & McAuliffe, 2017).

GMM clustering assumes each cluster follows a Gaussian distribution and that Gaussian components are sufficiently distinct to be statistically identifiable (McLachlan & Chang, 2004). Overlapping clusters with similar means and covariances can lead to identifiability issues, complicating parameter estimation. The model assumes observations are independent, simplifying the process by avoiding the need to account for correlations between data points across mixture components.

(8) $$p(x) = \sum\limits_{j = 1}^k {{\pi _j}} N(x|{\mu _j},{\Sigma _j}),\quad {w_{ij}} = {{{\pi _j}N({x_i}|{\mu _j},{\Sigma _j})} \over {\sum\nolimits_{l = 1}^k {{\pi _l}} N({x_i}|{\mu _l},{\Sigma _l})}}.$$

(9) $${\pi _j} = {1 \over n}\sum\limits_{i = 1}^n {{w_{ij}}} ,\quad {\mu _j} = {{\sum\nolimits_{i = 1}^n {{w_{ij}}} {x_i}} \over {\sum\nolimits_{i = 1}^n {{w_{ij}}} }},\quad {\Sigma _j} = {{\sum\nolimits_{i = 1}^n {{w_{ij}}} ({x_i} - {\mu _j}){{({x_i} - {\mu _j})}^T}} \over {\sum\nolimits_{i = 1}^n {{w_{ij}}} }}.$$

### Graph-based clustering: spectral clustering

Graph-based clustering involves transforming data into a graph format where nodes represent data points, and edges represent the relationships between these points. Relationships are quantified using similarity measures such as Euclidean distance, cosine similarity, or the Jaccard index for categorical data. These measures facilitate the construction of both directed and undirected graphs, with directed edges indicating directional relationships and undirected edges indicating mutual connections. Additionally, graphs can be weighted, where edge weights reflect the connection strength, or unweighted, where all connections are treated equally (Ester et al., 1996).

The primary objective in graph-based clustering is to partition the graph into clusters, or communities, where nodes within a cluster are more densely interconnected than those in different clusters. This aligns with community detection in network analysis, which aims to find groups of nodes (communities) that are more closely related to each other than to the rest of the network (Newman, 2004; Yang, Algesheimer & Tessone, 2016). Key graph properties leveraged include the node degree—the number of connections a node has—and the clustering coefficient, which measures the likelihood that two adjacent nodes are connected. These metrics provide insights into the cohesiveness of clusters (Newman, 2004; Von Luxburg, 2007).

Among graph clustering, spectral clustering, modularity maximization, and graph partitioning stand out due to their unique approaches to optimizing clusters. Spectral Clustering: Utilizes the eigenvectors of the graph’s Laplacian to form clusters, focusing on the graph’s global structure (Von Luxburg, 2007). Modularity Maximization: Aims to maximize the density of connections within clusters relative to what would be expected in a random edge distribution, thereby effectively identifying communities (Blondel et al., 2008). Graph Partitioning: Divides the graph into partitions by minimizing the number of inter-cluster edges and maximizing the internal cluster density.

(10) $${\mathrm{Minimize}}:\;{\bf{Tr}}({{{H}}^{{T}}}{{LH}})\quad {\mathrm{subject}}\;{\mathrm{to}}\;{{{H}}^{{T}}}{{H = I}}.$$

The time complexity of spectral clustering is primarily influenced by the computation of the similarity matrix and the subsequent eigenvalue decomposition. Constructing the similarity matrix typically requires $O({n^2})$ time, where $n$ is the number of data points (Yang, Algesheimer & Tessone, 2016). The most computationally expensive step is the eigenvalue decomposition, which has a time complexity of $O({n^3})$ in the worst case, though it can often be reduced to $O({n^2}\log n)$ with efficient algorithms for sparse matrices (Bishop, 2006). In terms of memory complexity, storing the similarity matrix requires $O({n^2})$ space (Murtagh & Contreras, 2012). Thus, spectral clustering can be computationally intensive and memory-demanding, particularly for large datasets (Fan et al., 2022).

### Autoencoders: deep embedded clustering (DEC)

Autoencoders are renowned for their ability to learn efficient representations of high-dimensional data by compressing data into a lower-dimensional latent space and then reconstructing it. This process captures essential features while filtering out noise, enhancing clustering algorithms by providing a streamlined and informative dataset, thus improving accuracy and interpretability (Goodfellow, Bengio & Courville, 2016). They handle non-linear relationships within data through non-linear activation functions and deep architectures, making them valuable in complex applications like anomaly detection (Guo et al., 2017).

Deep embedded clustering (DEC) builds on the strengths of autoencoders by integrating them with traditional clustering techniques. DEC starts by training an autoencoder to learn a meaningful latent representation of the data. The latent space representations are then used to initialize cluster centroids, typically with K-means. A clustering layer is added to the network, and the model is fine-tuned to jointly optimize both reconstruction and clustering losses, ensuring that the latent representations are conducive to forming well-defined clusters. Mathematical equations associated with DEC and t-SNE are as follows:

(11) $${\bf{z}} = f({\bf{x}}),\quad \widehat {\bf{x}} = g({\bf{z}}),\quad {L_{{\mathrm{recon}}}} = {1 \over N}{\sum\limits_{i = 1}^N {\left| {{{\bf{x}}_i} - {{\widehat {\bf{x}}}_i}} \right|} ^2}.$$

(12) $${q_{ij}} = {{{{(1 + {{\left| {{{\bf{z}}_i} - {{\mu }}j} \right|}^2}/\alpha )}^{ - {{\alpha + 1} \over 2}}}} \over {\sum {{j^\prime }} {{(1 + {{\left| {{\bf{z}}i - {{\mu }}{j^\prime }} \right|}^2}/\alpha )}^{ - {{\alpha + 1} \over 2}}}}}.$$

(13) $${p_{ij}} = {{q_{ij}^2/{f_j}} \over {\sum\nolimits_{{j^\prime }} {q_{i{j^\prime }}^2} /{f_{{j^\prime }}}}},\quad {f_j} = \sum\limits_i {{q_{ij}}}$$

(14) $${L_{{\mathrm{KL}}}} = \sum\limits_i {\sum\limits_j {{p_{ij}}} } \log {{{p_{ij}}} \over {{q_{ij}}}},\quad L = {L_{{\mathrm{recon}}}} + \gamma {L_{{\mathrm{KL}}}}.$$

The convergence time of DEC, depends on network architecture, data complexity, and optimization algorithms. Training involves iterative optimization, often computationally intensive for deep architectures, with complexity $O(I \cdot N \cdot D \cdot L)$ (Goodfellow, Bengio & Courville, 2016; Kingma & Welling, 2013). Techniques like stochastic gradient descent (SGD) can mitigate this cost and accelerate convergence. However, the non-convex optimization landscape poses challenges, with potential convergence to local optima or saddle points. Optimization techniques such as Adam or RMSprop help navigate this complex landscape but do not eliminate the risk of suboptimal convergence (Kingma & Ba, 2014; Duchi, Hazan & Singer, 2011).

In summary, DEC leverages autoencoders for dimensionality reduction and non-linear representation learning, integrating a clustering layer for joint optimization of reconstruction and clustering losses. This method addresses technical challenges and harnesses the full potential of DEC in clustering applications, making it a crucial tool in modern data analysis.

## Practical challenges of existing clustering methods

### Geometric constraints and density variations

Clustering algorithms group data points according to an inherent understanding of the underlying structure. These algorithms face certain limitations due to their reliance on modelling specific type of cluster distribution, due to design o their objective function. This focus on only a specific distrbution works, when you are modelling a similar distribution to their objective function or some complex distribution is a mixture of complex distributions.

**Figure 4:**
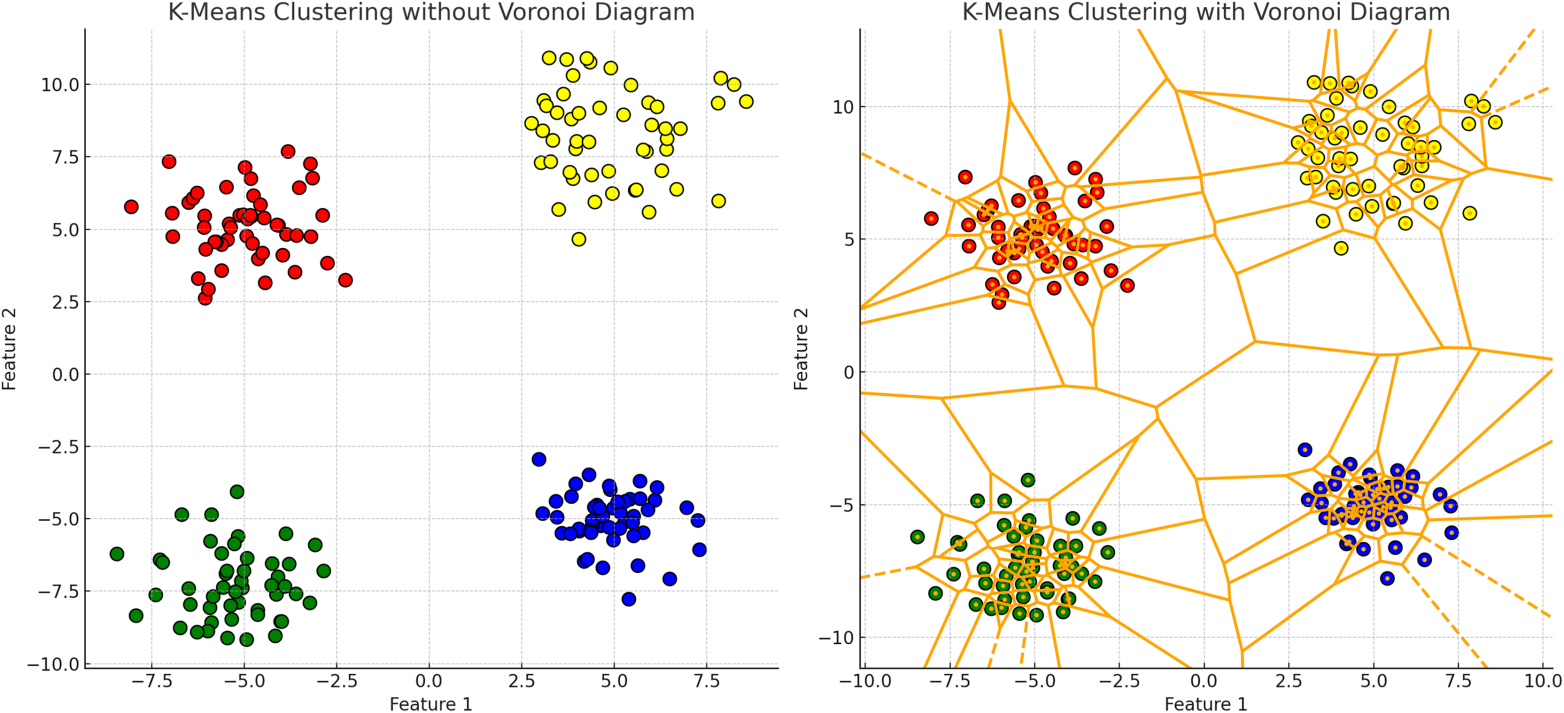
K-means clustering results with and without Voronoi diagram. The left plot demonstrates the K-means clustering result for a dataset consisting of four distinct clusters. The clusters are distributed across the plot as follows: the upper left quadrant contains a cluster of points tightly grouped around a centroid located approximately at coordinates (−5, 5); the upper right quadrant features another cluster centered around coordinates (5, 5); the lower left quadrant includes a cluster centered near coordinates (−5, −5); and the lower right quadrant has a cluster centered around coordinates (5, −5). In the right plot, the same K-means clustering result is displayed with the addition of a Voronoi diagram. The Voronoi diagram partitions the plane into regions where each region contains all the points closest to a particular cluster centroid. The partitioning lines delineate these regions. The centroids of the clusters are marked within each region, demonstrating the areas of influence each centroid has over the surrounding points.

**Figure 5:**
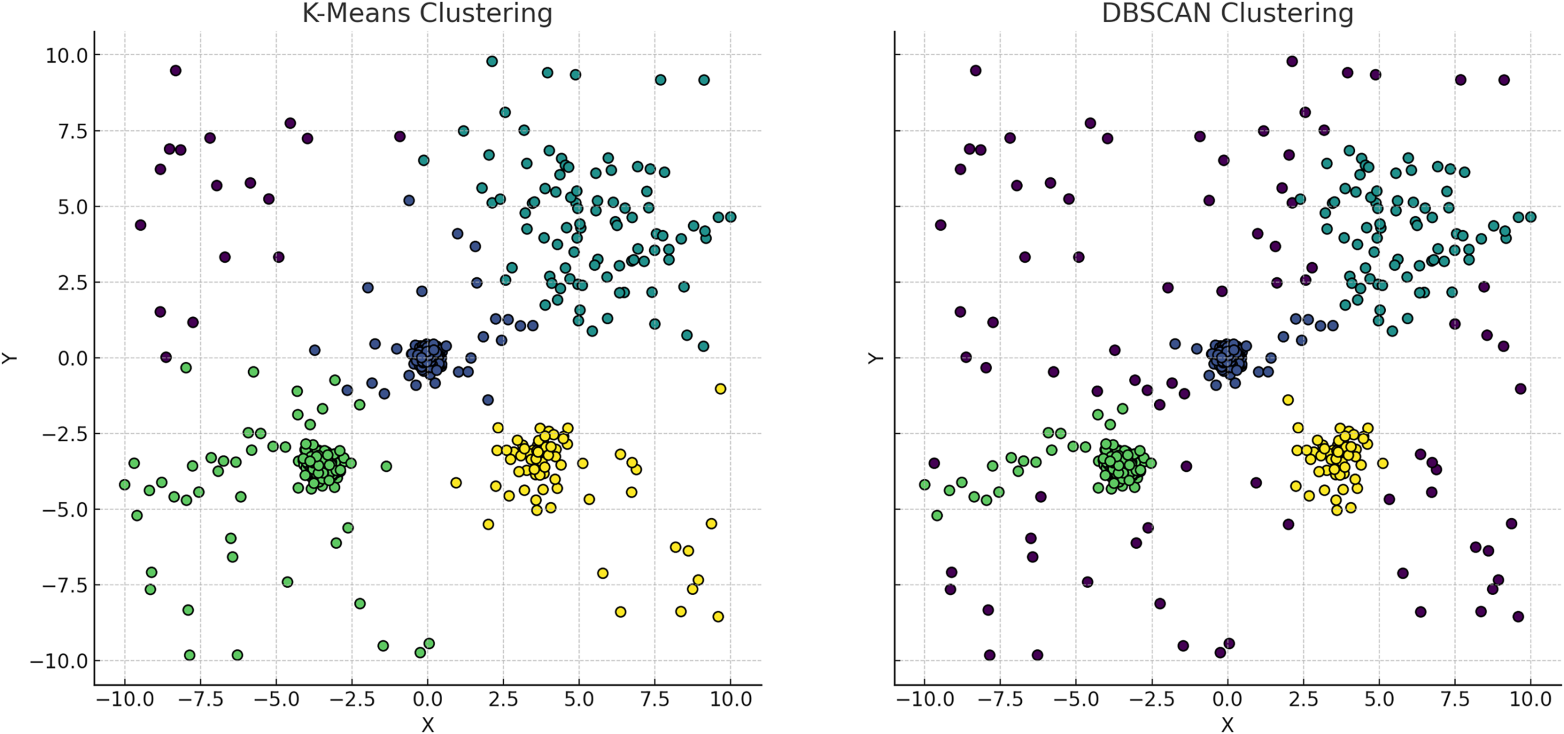
Comparative visualization of the K-means and DBSCAN clustering algorithms. The left plot, representing K-means clustering, demonstrates how it imposes spherical cluster shapes and evenly distributes data points among a predefined number of clusters, which may not align with the natural groupings within the data. Conversely, the right plot, representing DBSCAN clustering, effectively identifies clusters based on data density, accommodating clusters of varied shapes and sizes. This capability of DBSCAN to adapt to data distribution without pre-specifying the number of clusters is particularly advantageous for datasets with complex spatial relationships and varying densities, highlighting its superiority in scenarios where the distribution of data points is non-uniform or when the presence of noise and outliers is significant.

**Figure 6:**
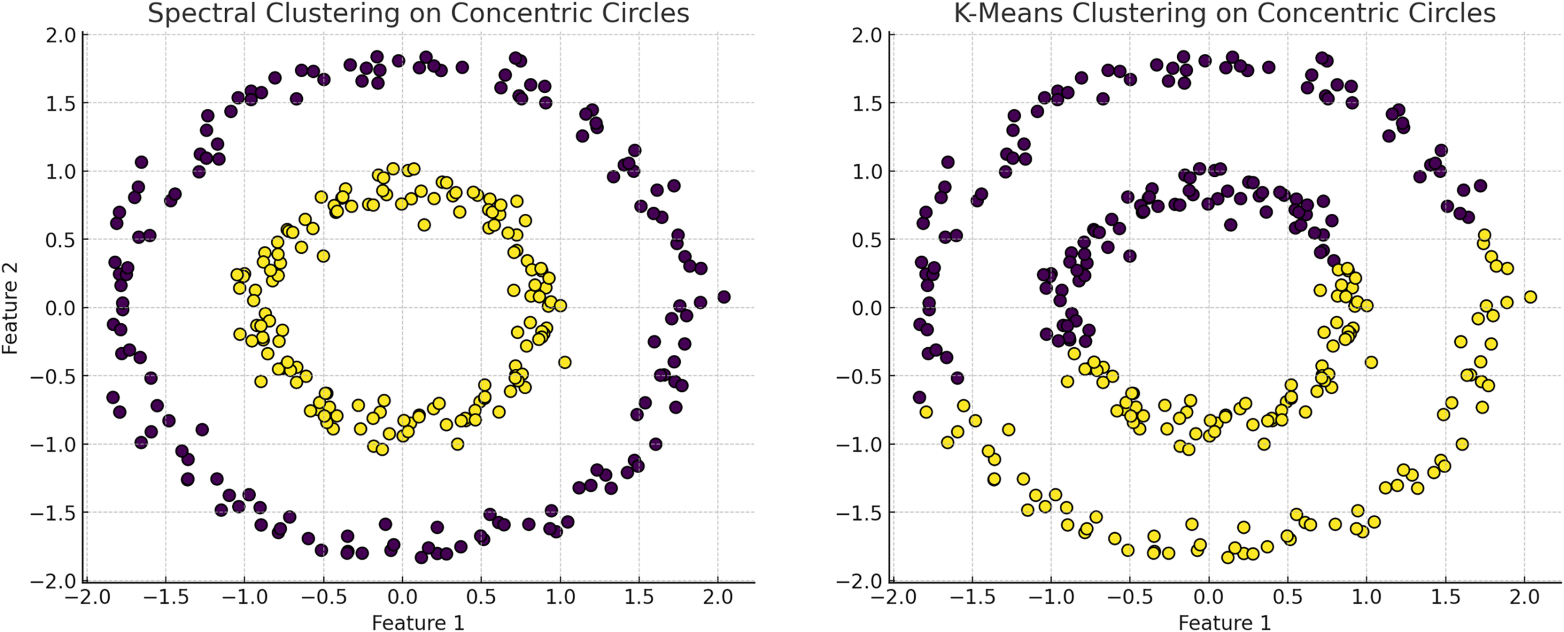
The plot has been generated using simulated data from sklearn.datasets ‘make circles’. Efficiency of spectral clustering *vs*. K-means on concentric circles: The left plot demonstrates spectral clustering’s capability to segregate non-linearly separable structures, effectively clustering concentric circles. Conversely, the right plot illustrates K-means’ limitations, misclassifying similar datasets due to its assumption of globular cluster shapes. This comparison underscores spectral clustering’s adaptability to complex data geometries, outperforming K-Means which struggles with non-spherical distributions.

- **K-means clustering** partitions data points into k clusters by minimizing the WCSS, assuming clusters are spherical and isotropic, illustrated by the voroni diagram on Fig. 4 (Lipson & Siegelmann, 2000). This makes it ineffective for non-spherical or elongated shapes. Centroids shift towards the mean and move to the densest regions, leading to challenges with non-spherical shapes or varying densities, merging distinct clusters or fragmenting cohesive ones. Algorithms like fuzzy c-means and fuzzy K-means, allowing partial membership to multiple clusters, are more suitable for datasets with overlapping characteristics (Xu & Wunsch, 2005; Nayak, Naik & Behera, 2015.
- **Hierarchical clustering** relies heavily on linkage criteria and distance metrics, influencing the shapes and sizes of the resulting clusters. This method struggles with non-convex shapes or clusters of varying densities due to its reliance on pairwise distances. This leads to the formation of elongated or chain-like clusters that may not accurately represent the underlying data. These geometric limitations can distort relationships in datasets with varying densities, leading to inappropriate mergers or divisions, especially in irregular cluster shapes.
- **DBSCAN** identifies clusters based on dense regions separated by sparse areas, accommodating clusters of arbitrary shapes and sizes, which is ideal for non-convex clusters, illustrated in Fig. 5, cmparing kmeans and dbscan. However, DBSCAN struggles with clusters of varying densities and irregular shapes (Bataineh & Alzah, 2023). The algorithm’s reliance on parameters like the neighborhood radius and minimum number of points can lead to suboptimal results for clusters with different densities (Wang, Lu & Rinaldo, 2019). Additionally, DBSCAN may improperly separate clusters that are too close or have varying densities, resulting in merged or fragmented clusters.
- **GMM** is able to captures varying cluster shapes by adjusting the Gaussian components parameters. This soft clustering approach adapts well to complex distributions and provides a probabilistic measure of cluster membership, which is richer in interpretation than hard assignments (Fraley & Raftery, 2002). However, GMM struggles with identifying clusters with overlapping regions. The reliance on Gaussian distribution limits it’s effectiveness when the data violates this assumption, and the fixed covariance structure may fail to accurately reflect the true spread within each cluster (Knief & Forstmeier, 2021).
- **Spectral clustering** uses the eigenvalues of the similarity matrix of the data to perform dimensionality reduction before clustering in lower dimensions. This method is effective for identifying clusters that are not necessarily globular but can struggle with varying densities, illustrated on Fig. 6. The construction of the similarity matrix and the subsequent eigenvalue decomposition may not always capture the true distances and densities within the data.

### Sensitivity to initialization conditions

Regardless of the choice of clustering algorithms, initialization parameters influence the efficacy and outcome of the analysis. These parameters, established before the algorithm’s operation, not only guide the computational process but also significantly affect the quality of the final clustering results (Hastie et al., 2009). Choosing optimal initialization parameters is crucial as poor selections can lead to convergence issues, unstable results, and inaccurate representations of data groupings (Gul & Rehman, 2023).

- **K-means clustering** is particularly sensitive to the initial parameter values, especially the positions of cluster centroids, highlighted in Fig. 1. Centroids significantly influence cluster memberships and the discovery of a dataset’s inherent structure. Inappropriately placed initial centroids can result in poor clustering performance, misrepresenting the underlying patterns of the data. Since K-Means iteratively adjusts centroids towards the mean of assigned points, their initial positions are crucial for effective and efficient convergence, with incorrectly chosen centroids potentially leading to slow convergence or incorrect solutions (Arthur, 2007; Jiacai & Ruijun, 2010).
- **Hierarchical clustering** build clusters based on data point proximity or connectivity, often starting each point as its cluster and merging them iteratively based on a specific linkage criterion. The initial setup and the linkage criteria selected (*e.g*., single, complete, average linkage) can profoundly impact the clustering path and, consequently, the final outcomes (Hastie et al., 2009). These models are inherently sensitive to initial conditions because once a connection between points is established, it cannot be altered; thus, early decisions significantly influence the entire clustering structure.
- **DEC** The performance of autoencoders is highly influenced by initial weights. Poor initialization can lead to suboptimal local minima, affecting clustering quality. Effective initialization techniques, such as pre-trained weights and advanced algorithms like Xavier (Glorot) and He initialization, can enhance robustness (Glorot & Bengio, 2010). These techniques improve convergence behavior and the quality of learned representations by providing better starting points and considering the size of previous layers and activation functions.
- **DBSCAN** is fundamentally resiliant to initial parameter settings. These algorithms focus on identifying dense regions separated by areas of lower density, which allows them to be less influenced by outliers and capable of detecting clusters of various shapes and sizes naturally present in the data (Xu & Tian, 2015). This attribute is particularly advantageous in applications with complex data structures where traditional clustering methods might fail.

### Overcoming bias in cluster analysis

**Figure 7:**
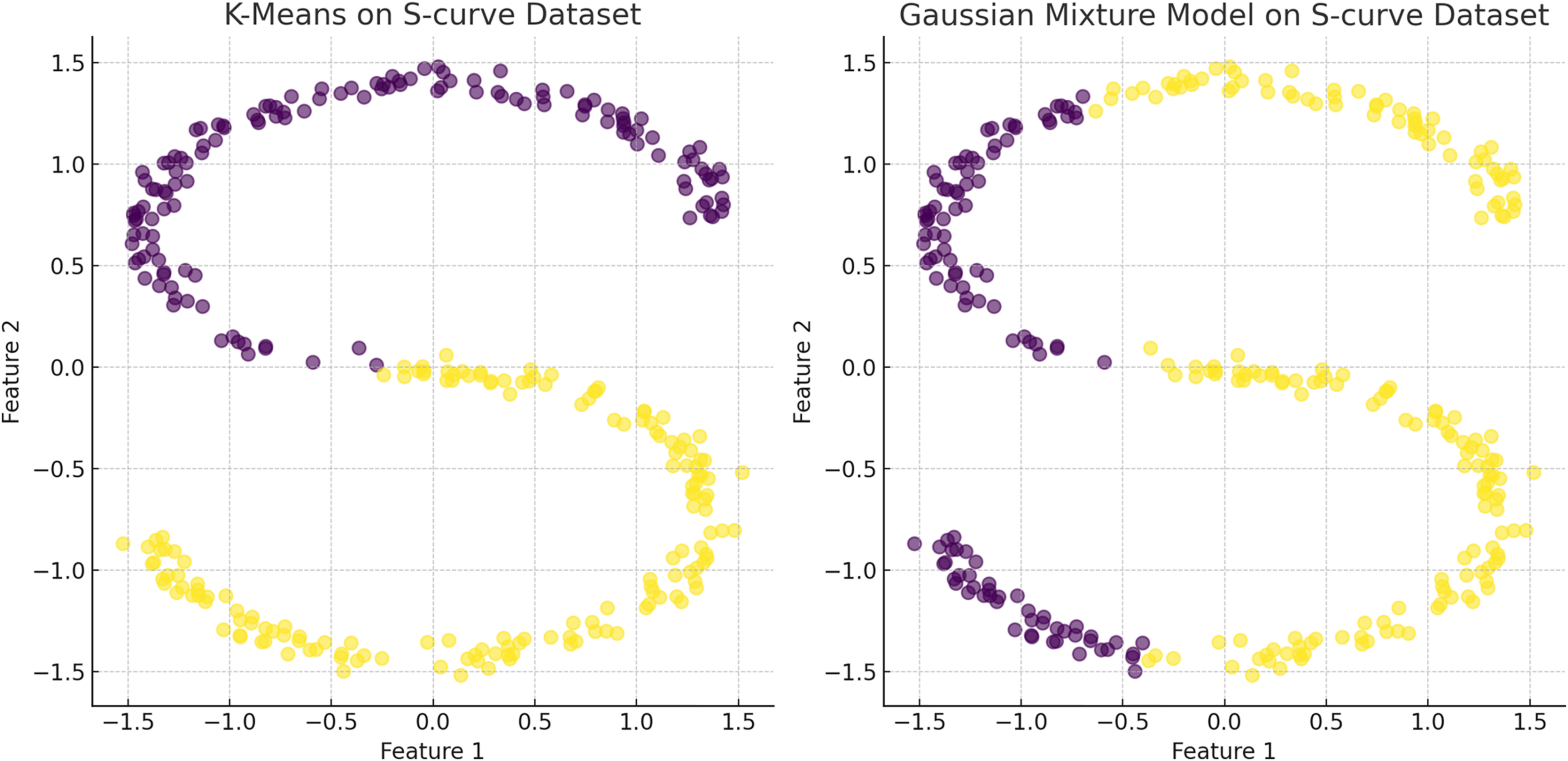
The plot has been generated using simulated data from sklearn.datasets ‘make s curves’. Comparative analysis of K-means and Gaussian mixture model on an S-Curve Dataset: The left plot shows K-means’ limitations with linear segmentation that overlooks the dataset’s intrinsic curvature, resulting in an oversimplified cluster representation. In contrast, the right plot demonstrates how the Gaussian mixture model leverages a probabilistic approach for soft clustering, which adapts flexibly to the S-curve’s continuous nature and density variations, illustrating its effectiveness in handling non-linear data distributions.

In clustering algorithms, the absence of ground truth data necessitates reliance on initial assumptions, such as the predetermined number of clusters ( $k$), which can significantly bias the outcome towards these initial settings (Jain, 2010; Kaufman & Rousseeuw, 2009). This scenario is particularly evident in K-means, where the algorithm’s objective to minimize intra-cluster variance directly correlates to the specified $k$, potentially constraining the analysis within an arbitrary framework that might not accurately represent the underlying data structure (Arthur, 2007; Tibshirani, Walther & Hastie, 2001). Alternative clustering approaches like DBSCAN and distribution based clustering provide a less biased exploration of data groupings, as they do not require a predefined $k$. DBSCAN, for *e.g*., delineates clusters based on the density of data points, allowing for the identification of clusters of varying sizes and shapes without the constraint of specifying $k$, illustrated in the Fig. 5 (Ester et al., 1996; Fraley & Raftery, 2002). Distribution-based clustering assumes data originates from a mixture of underlying statistical distributions, with the cluster count inferred directly from the data, reducing bias towards any predetermined number of clusters, illustrated in the Fig. 7. Addressing the bias introduced by the specification of $k$ involves employing evaluation metrics such as the silhouette coefficient or the Calinski-Harabasz index, which objectively assess the clustering quality for different $k$ values (Rousseeuw, 1987). Leveraging these metrics helps identify a cluster count that more naturally aligns with the data. Additionally, the strategic application of domain knowledge, while beneficial, must be approached with caution to prevent the introduction of further biases. Ultimately, the challenge lies in mitigating the accidental bias towards initial assumptions, a task that necessitates a nuanced approach combining algorithmic flexibility, informed parameter selection, and the judicious use of evaluation metrics. By embracing these strategies, it becomes possible to reveal the data’s true structure, leading to more authentic and insightful clustering outcomes.

### The curse of dimensionality in clustering algorithms

The curse of dimensionality refers to the challenges that arise as the number of dimensions increases. Challenges include increased data sparsity and diminished effectiveness of traditional distance metrics, severely impacting the performance of clustering algorithms, particularly centroid-based and connectivity-based models. As dimensionality grows, data points tend to spread out, leading to a sparse distribution which complicates the identification of meaningful clusters and undermines the reliability of distance measures (Han, Pei & Tong, 2022; Keogh & Mueen, 2017). This sparsity makes traditional distance measures less effective, as the distinction between the nearest and farthest points diminishes, challenging the clustering algorithms ability to group data based on similarity (Bellman & Kalaba, 1959).

- **K-means** clusters face difficulties with the dispersion of data points in high-dimensional spaces. These are are spherical and exhibit uniform variance across all dimensions becomes increasingly untenable. This assumption leads to inaccuracies in defining cluster boundaries and updating centroids, which are critical to the K-means algorithm (Arthur, 2007; Steinbach, Ertöz & Kumar, 2004).
- **Hierarchical clustering:** These models, which include HAC, struggle with the empty space phenomenon where the vast distances in high-dimensional spaces mislead the clustering process, often resulting in fragmented and poorly defined cluster (Steinbach, Ertöz & Kumar, 2004).
- **DBSCAN:** These are generally more robust against the curse of dimensionality due to their focus on density rather than distance, they still face challenges in accurately identifying dense regions amid the overall sparsity. Their effectiveness hinges on appropriately calibrated density thresholds, which can be complex to adjust in high-dimensional spaces (McInnes, Healy & Astels, 2017; Li et al., 2019).
- **DEC** tackles the curse of dimensionality by projecting high-dimensional data into a lower-dimensional latent space, capturing significant features. However, training on high-dimensional data remains computationally intensive, and the success of dimensionality reduction depends on the architecture and training process. If not properly tuned, the latent space may still exhibit high-dimensional characteristics, leading to inefficiency. Variational autoencoders (VAEs) use probabilistic approaches to create more structured latent spaces, mitigating some high-dimensional challenges. Nonetheless, ensuring encoded representations effectively reduce dimensional complexity without losing critical information remains challenging.
- **Spectral clustering** deal with the curse by constructing similarity graphs. The increased computational complexity and dilution of meaningful distance complicate maintaining effective node connections and clearly delineating cluster boundaries in high-dimensional spaces (Sui et al., 2020).

### Difficulty dealing with noisy data

Effectively managing noise in datasets is crucial for accurate clustering, as unaddressed noise can undermine results and lead to incorrect interpretations (Ben-David & Haghtalab, 2014; Han, Pei & Tong, 2022). Identifying and mitigating noise enhances the reliability and validity of clustering outcomes, clarifying the dataset’s quality and aiding in the strategic selection and adjustment of clustering methods and parameters. Noise can distort cluster boundaries, increase within-cluster variance, and form erroneous clusters, complicating the identification of the true number of clusters and obscuring the data’s actual structure (Han, Pei & Tong, 2022; Xu & Tian, 2015). Clustering algorithms react to presence of noise differently. Understanding these dynamics is crucial for applying the most suitable clustering methodology to noisy data, ensuring more reliable and insightful analytical results.

**Figure 8:**
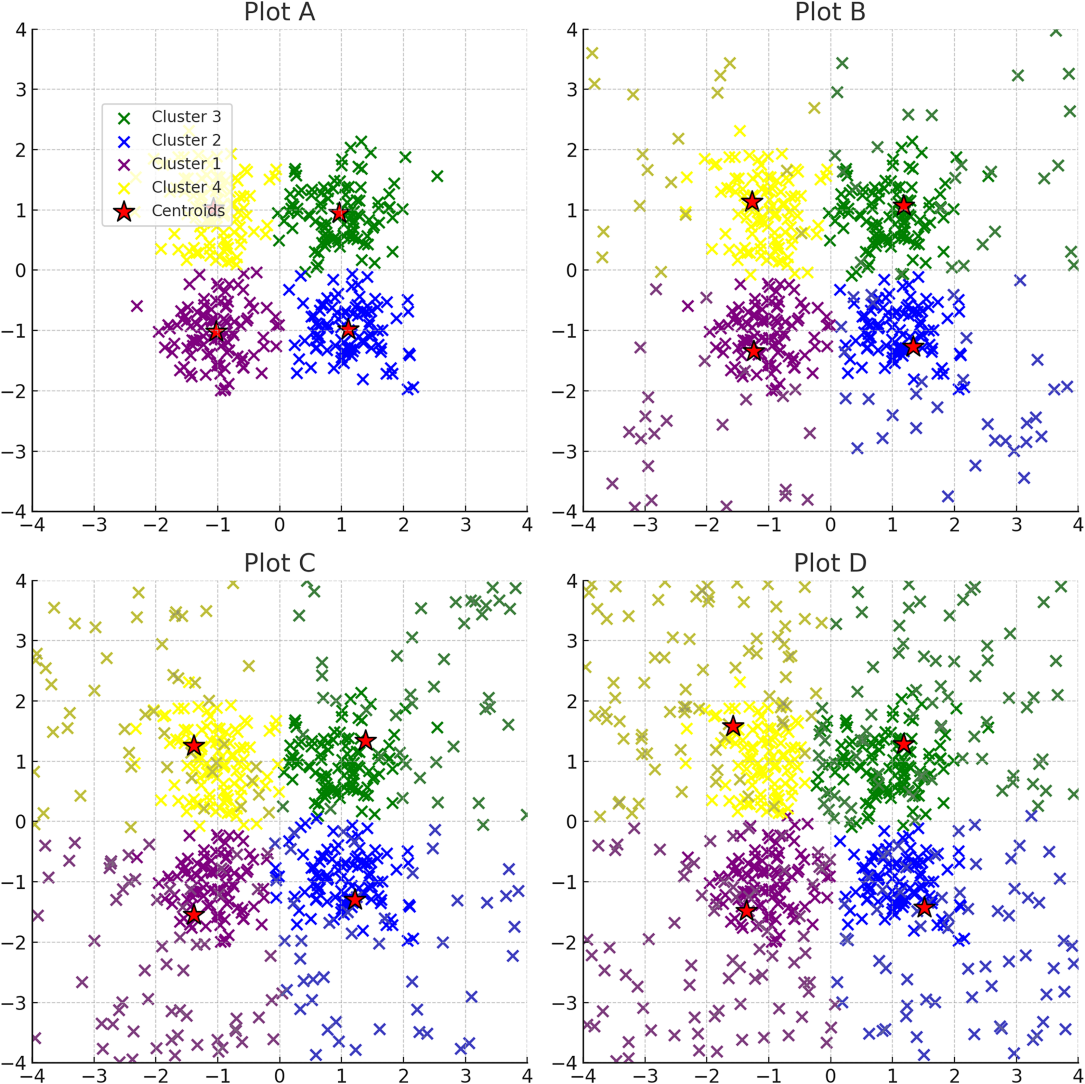
Progressive addition of noise to clusters: plots A through D depict the effect of incrementally increasing noise on a dataset originally consisting of four clusters. Noise points are shown in the same color as the original clustering section colours. The red stars indicate the centroids of the clusters after applying K-means clustering.

**Figure 9:**
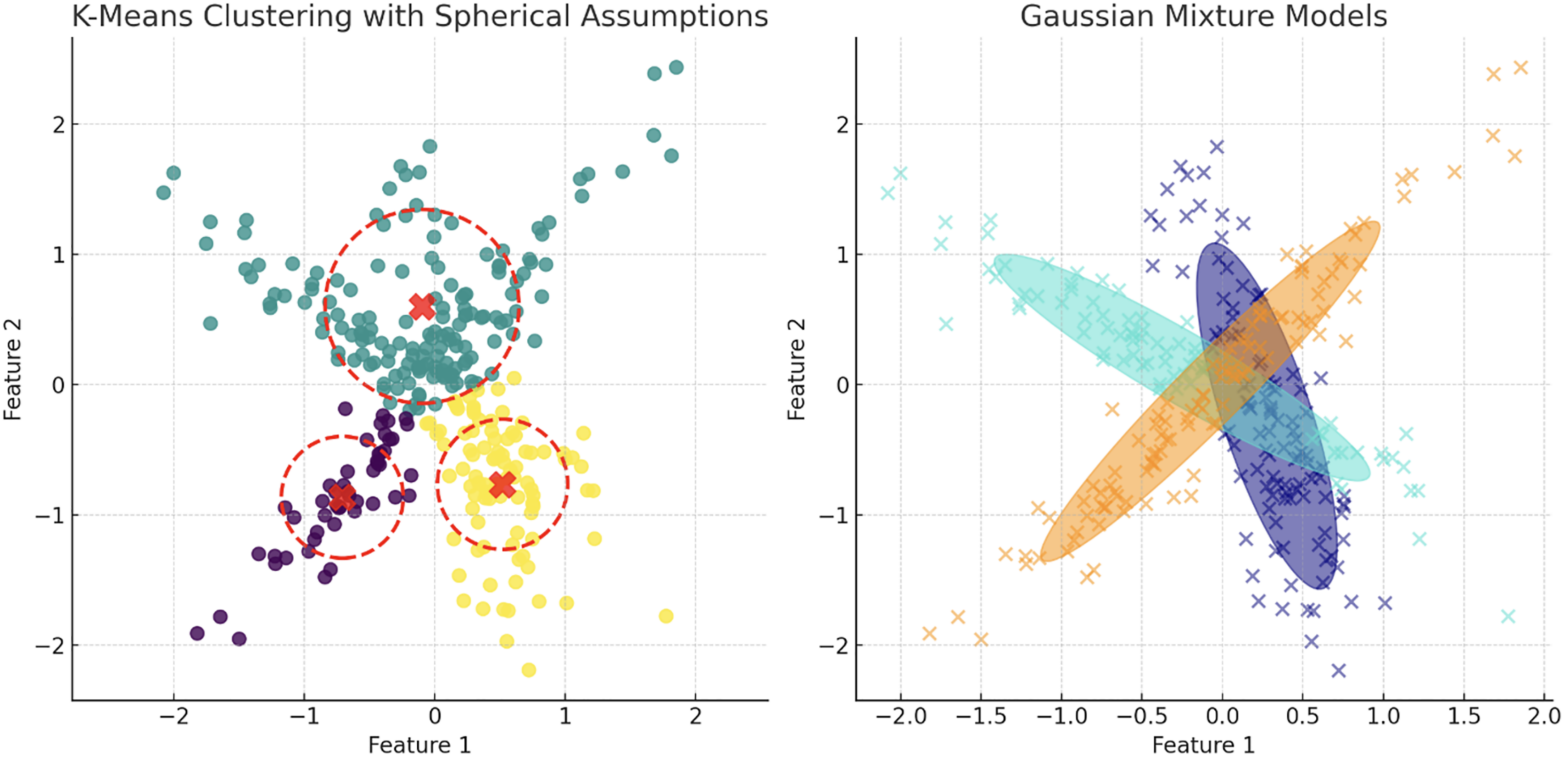
Contrasting clustering algorithms on multimodal data: the left plot illustrates K-means clustering with spherical assumptions, highlighting its limitations through misaligned centroids and overlapping clusters due to its inability to account for non-spherical distributions. The right plot displays GMM, effectively capturing the underlying data structure with ellipsoidal components that conform to the data’s true distribution, showcasing GMM’s flexibility in modeling complex cluster shapes and orientations.

- **K-means** face a dual challenge when noise is present: centroids can be misleadingly dragged by noise points, and the algorithm’s criteria for cluster cohesion are compromised, often resulting in an overestimation of cluster numbers or inclusion of noise points in clusters. K-means clustering is particularly vulnerable to noise and outliers, illustrated in Fig. 8; as the calculation of centroids is heavily influenced by extreme values, which can significantly skew the clustering results.
- **Hierarchical clustering** sensitivity varies with the linkage criterion employed, where noise can cause premature linkage or prevent meaningful clusters from merging at the correct scale. Density-based methods, focusing on local density rather than global structure, inherently ignore noise points during cluster formation, making them suitable for datasets with significant noise. However, the choice of $\varepsilon$ (the neighborhood radius for density calculation) is critical; too small a value might ignore meaningful points as noise, while too large a value could merge distinct clusters (Ester et al., 1996; Le-Khac et al., 2010).
- **DEC** although effective at filtering some noise, can struggle if training data is not well-preprocessed. Noise can distort the latent space representations, degrading clustering performance. Regularization techniques such as dropout and L2 regularization can help mitigate noise impact. Dropout randomly omits neurons during training, encouraging the network to learn robust features. L2 regularization adds a penalty term to the loss function proportional to the square of the weights, preventing overfitting. Denoising autoencoders, trained to reconstruct the original input from a corrupted version, enhance robustness against noise by learning to ignore irrelevant variations.
- **GMMs**, by design, are less susceptible to outliers because they model clusters using probability distributions, which theoretically provide some robustness against anomalies, illustrated in Fig. 9. However, the fundamental assumption that data points are derived from a Gaussian mixture does not adequately address datasets characterized by heavy-tailed distributions or outlier populations.
- **Spectral clustering** Noise can introduce incorrect edges or affect edge weights, misleading the model about the strength or nature of relationships between points. In high-dimensional spaces, graphical models can become overly complex, with noise adding spurious connections or diluting important ones. The initial construction of the graph (*e.g*., node connections) is crucial, as noise can misrepresent the data’s structure, affecting subsequent clustering steps.

### Dealing with mixed feature types

Handling datasets with mixed feature types, encompassing both categorical and numerical variables, presents a significant challenge in clustering. Categorical variables classify qualitative attributes into discrete groups, whereas numerical variables measure attributes on a continuous scale (Azen & Walker, 2021). This fundamental difference complicates the application of traditional distance metrics, such as Euclidean for numerical data and Jaccard for categorical data, within a unified analytical framework (Han, Pei & Tong, 2022). Integrating these disparate data types into a single clustering analysis often introduces distortions that undermine the validity of the outcomes. Using standard distance metrics, such as Euclidean distance, in clustering algorithms often leads to inaccurate proximity representations, especially with numerically encoded categorical variables (Mehta, Bawa & Singh, 2020). These metrics treat numerical codes as representing linear intervals, imposing a continuous metric on inherently discrete categories, which distorts the perceived proximity between data points and compromises the integrity of the derived clusters.

Additionally, integrating variables on (categorical *vs*. numerical) introduces biases in feature weighting within clustering algorithms. Standardizing data and implementing customized weighting schemes are typical strategies to counteract these biases, but they often fail to eliminate the disproportionate influence exerted by variable types. Consequently, the resulting clusters might over-emphasize categorical variables or under-represent numerical variations, leading to reduced interpretability and diminished accuracy of the clustering results. These issues are problematic for the field of clustering because they lead to invalid conclusions about the underlying structure of the data. Misrepresentations and biases can result in clusters that do not accurately reflect the true relationships and patterns within the data, compromising the reliability and applicability of clustering results, which are crucial for tasks such as data exploration, pattern recognition, and decision-making in various domains (Huang, 1998).

### Absorption of smaller clusters due to imbalanced cluster size

Clustering algorithms often favor the formation of uniformly sized clusters, potentially misrepresenting the underlying data. This bias can obscure smaller clusters within imbalanced datasets, where larger clusters dominate (Zhan et al., 2021; Lin et al., 2017). The optimization criteria may inadvertently prioritize larger clusters, and methods using global thresholds or density estimates struggle to detect subtle differences in smaller clusters (Singh & Dhall, 2018; Tsai et al., 2019). The selection of a clustering technique should be guided by the specific characteristics and requirements of the data, especially when dealing with imbalanced clusters. Understanding these nuances is essential for achieving accurate clustering outcomes.

- **Hierarchical clustering** build models based on distance connectivity. These methods do not assume clusters to be of a particular geometry or size. HAC creates clusters by either iteratively merging the furthest or most dissimilar points. This method is quite flexible in handling clusters of varying sizes and shapes, as the clustering process is solely based on the distance between data points or pre-existing clusters, not on pre-assumed cluster distribution (Guha, Rastogi & Shim, 1998).
- **K-means** begins by initializing k centroids and iteratively assigns points to the nearest centroid, followed by recalculating the centroids (Celebi, Kingravi & Vela, 2013). It assumes the cluster center accurately represents the cluster, an assumption that may not hold for imbalanced clusters, particularly if they are in close proximity to another centroid (Duan et al., 2020). Such methods may face difficulties with varying cluster sizes, as smaller clusters could be challenging to identify and may be mistakenly grouped with larger clusters, as the centroids of larger clusters attract boundary points of smaller clusters, potentially obscuring their distinct identities (Jain & Dubes, 1988; Arthur, 2007).
- **DEC** face significant challenges when dealing with imbalanced data. They tend to prioritize features representing larger clusters, leading to poor reconstruction for smaller clusters due to the dominance of majority class characteristics in the latent space (Shen et al., 2018). The reconstruction loss, often driven by the majority class, exacerbates this issue by neglecting the nuances of minority clusters (Buda, Maki & Mazurowski, 2018). This bias persists even in clustering within the latent space, making it difficult to identify smaller clusters accurately (Guo et al., 2017).
- **DBSCAN** methods are particularly well-suited for handling clusters of varying sizes and shapes, as they define clusters as areas of high density separated by areas of low density. Unlike methods that rely on pre-defined centroids or connections, density based algorithms can identify clusters of arbitrary shapes and sizes (Ester et al., 1996). The main advantage of density based methods lies in their flexibility to adapt to the structure of the data without being biased toward any specific cluster sizes or shapes, making them well-suited to handle imbalanced cluster distributions. Additionally, these methods are adept at identifying outliers or noise points that do not belong to any cluster.

### Clustering challenges in big data and streaming environments

The proliferation of big data and streaming environments is transforming data analytics, introducing unique challenges for clustering algorithms. These algorithms now require flexibility and adaptability to effectively handle dynamic data. Maintaining clustering models effectiveness postdeployment, rigorous monitoring systems are essential for early detection of performance declines. This allows timely interventions such as model retraining, parameter optimization, or adopting new algorithmic strategies to address changes in data characteristics (Tsai et al., 2019; Silva et al., 2013; Kriegel, Kröger & Zimek, 2009).

- **K-means and GMM** K-Means struggle with evolving data distributions due to their reliance on static centroid positions, which may become unrepresentative as data evolves, leading to performance degradation and misclustering (Cao et al., 2006). Since GMM assume data points are generated from known distributions. if there are fluctuations in these underlying distributions, it could invalidate the model, affecting clustering accuracy (Zivkovic, 2004)
- **Hierarchical clustering** Methods rely on constructing dendrograms based on distance metrics that may become obsolete as relationships within the data change, resulting in outdated hierarchical structures (Murtagh & Contreras, 2012).
- **DBSCAN and spectral clustering** DBSCAN depends on predefined density thresholds for cluster identification, face challenges when there are shifts in the overall data density landscape, leading to potential misidentification of clusters (Campello, Moulavi & Sander, 2013). Spectral clustering construct similarity graphs to identify clusters can suffer when the inherent data relationships they rely on evolve, undermining the stability of the resultant clusters (Liu & Han, 2018).
- **DEC** learn compact, non-linear feature representations which are useful for clustering tasks. However, they face challenges in maintaining up-to-date latent space representations as new data continually arrives. This causes the initially learned representations to become outdated, reducing clustering effectiveness.

## Solutions for overcoming clustering limitations

### Dimensionality reduction

Dimensionality reduction techniques help uncover underlying patterns within data, especially as modern datasets grow in complexity and dimensionality. Traditional clustering approaches often struggle to provide meaningful insights in such scenarios. Dimensionality reduction offers a solution to this challenge, transform high-dimensional data into a more manageable form while maintaining its inherent structure. This preprocessing step enables clustering algorithms to operate more effectively, revealing the natural groupings and relationships within the data. These techniques not only help in managing the computational complexity of high-dimensional data but also contribute to more accurate and insightful clustering results.

- **Principal component analysis (PCA)**: serves as a fundamental technique for reducing the dimensionality of large data sets, enhancing interpretability while minimizing information loss. It achieves this by transforming the original variables into a new set of variables—eigenvectors, which are orthogonal to each other and ordered by the amount of variance they capture from the data. This method is particularly effective in reducing the noise and complexity of high-dimensional data, making the clustering process more efficient and robust (Jolliffe, 2002).
- **t-distributed Stochastic Neighbor Embedding (t-SNE)**: This is a non-linear technique that excels in visualizing the clustering of complex datasets by reducing dimensions while maintaining local data structures. It is particularly adept at revealing patterns and clusters in data that are not apparent in higher dimensions (van der Maaten & Hinton, 2008).
- **DEC:** Autoencoders are trained to reconstruct input data, capturing underlying patterns and relationships. The encoder maps input data to a lower-dimensional latent space, effectively compressing the information into a compact representation (Goodfellow, Bengio & Courville, 2016). This helps identify the most informative features that contribute to the data’s structure and variability (Kingma & Welling, 2013; Goyal & Ferrara, 2018). By learning nonlinear transformations, autoencoders can capture intricate patterns and relationships not apparent in the original feature space, which is useful for datasets with nonlinear structures or complex feature interactions (Goodfellow, Bengio & Courville, 2016).
- **Uniform Manifold Approximation and Projection (UMAP):** assumes data is uniformly distributed on a locally connected manifold and each point can be accurately represented by its nearest neighbors (McInnes, Healy & Melville, 2018; Becht et al., 2019). UMAP constructs a high-dimensional graph of the original data using fuzzy simplicial sets to retain both local and global structures (van der Maaten & Hinton, 2008). Connections between points in the graph are weighted based on distance, with closer points having stronger connections. The goal of UMAP is to accurately layout this high-dimensional graph in a lower-dimensional space (McInnes, Healy & Melville, 2018).

### Overcoming initialization challenges

The performance of many clustering algorithms, especially those like K-means that are sensitive to initial conditions, can be significantly influenced by how they are initialized. Poor initialization can lead to suboptimal clustering results, with algorithms potentially converging to local minima rather than the global optimum. To mitigate this, several robust strategies have been developed to enhance initialization and, consequently, the overall robustness and accuracy of clustering outcomes.

- **Multiple initialization:** The method of multiple initializations involves running the clustering algorithm multiple times with different random starting conditions. This technique broadens the potential solutions, increasing the chances of achieving a near-global optimum by avoiding local minima. Despite its computational demands, it is highly effective and commonly used, with the best result often selected based on the lowest sum of squared distances within clusters.
- **Informed initialization:** Informed initialization methods leverage domain knowledge or preliminary data analysis to strategically select initial settings, enhancing clustering effectiveness. *E.g*. K-means++ improves initial separation of cluster centroids promoting better convergence and reducing likelihood of settling into local optima. These methods incorporate prior information to mitigate the common issue of sensitivity to initial conditions in various clustering frameworks (Arthur, 2007; Bahmani et al., 2012).
- **Early stopping mechanisms**, which halt the algorithm when no significant improvement is observed in metrics such as the silhouette score or inertia over several iterations, prevent overfitting and save computational resources. This method optimizes resource use and safeguards the model from potential degradation due to excessive processing.
- **Tuning key hyperparameters:** like the number of clusters and the choice of distance metric profoundly influences initial conditions and, by extension, the clustering results. Methods such as grid search are employed to systematically explore parameter spaces and identify optimal settings, effectively reshaping the initialization landscape to favor convergence to a globally optimal solution.
- **Warm starting:** uses parameters from previous runs as the basis for new iterations, proves highly beneficial, particularly in dynamic environments where data characteristics subtly evolve. This approach accelerates convergence and enhances the efficiency of the algorithm, making it ideal for applications that require frequent updates.

By integrating advanced strategies such as multiple initializations, informed techniques, early stopping, parameter tuning, and warm starts, a comprehensive framework is established to address the challenges associated with initial parameter sensitivity in clustering algorithms. These empirically supported methods not only ensure convergence towards more globally optimal solutions but also refine the accuracy and interpretability of the clusters, thereby bolstering confidence in the decisions derived from these models.

### Ensembling and balanced loss functions

Conventional clustering algorithms often exhibit bias toward larger, dominant clusters, overlooking smaller, minority groups. This article addresses this issue by proposing a blend of ensemble clustering methodologies and balanced loss functions. Ensemble clustering leverages multiple algorithms to enhance robustness, while balanced loss functions mitigate bias by assigning appropriate weights to smaller clusters, ensuring fair and representative clustering outcomes.

- **Ensemble clustering** enhances the stability of clustering results by combining multiple base clusterings. This involves generating diverse base cluster configurations and integrating them through consensus functions. Deep learning-based clustering ensembles are particularly effective for high-dimensional data. By selecting diverse, high-quality base clusterings and refining them through advanced consensus functions, ensemble methods significantly improve overall clustering performance and resilience against biases inherent in individual models (Strehl & Ghosh, 2002).
- **Balanced loss functions** enhance the influence of smaller, underrepresented clusters within the optimization framework. Inspired by the Synthetic Minority Over-sampling Technique (SMOTE) (Chawla et al., 2002), these methods adjust the contribution of data points to the loss function without physically augmenting the dataset. By assigning higher weights to smaller clusters, the model’s sensitivity to these minority groups is enhanced. This prevents predominant features or cluster sizes from skewing the clustering results (Kriegel, Kröger & Zimek, 2009). Balanced loss functions are:
- **Class-balanced loss:** Adjusts the loss contributions based on the frequency of each cluster, ensuring underrepresented clusters significantly impact the overall loss.
(15) $${L_{{\mathrm{balanced}}}} = {1 \over N}\sum\limits_{i = 1}^N {{{{L_i}} \over {{\mathrm{frequency}}({c_i})}}}.$$
- **Focal loss:** Addresses class imbalance by down-weighting the loss assigned to well-classified examples and focusing more on hard, misclassified examples.
(16) $${L_{{\mathrm{focal}}}} = - \alpha {(1 - {p_t})^\gamma }\log ({p_t}).$$
- **Reweighted loss:** Assigns different weights to clusters based on their sizes, giving higher weights to smaller clusters.
(17) $${L_{{\mathrm{reweighted}}}} = \sum\limits_{i = 1}^N {{w_{{c_i}}}} {L_i}.$$
- **Cost-sensitive loss:** Incorporates a cost matrix defining the penalty for misclassifications, giving higher penalties to misclassifications involving minority clusters.
(18) $${L_{{\mathrm{cost - sensitive}}}} = \sum\limits_{i = 1}^N {{C_{{y_i},{{\hat y}_i}}}} \cdot {L_i}.$$

Integrating ensemble learning and balanced loss functions enhances cluster fairness and robustness. Ensemble clustering combines models trained on distinct data subsets or initialized with varying parameters, capturing a broader spectrum of patterns. This diversity moderates the impact of disparate clusters and mitigates inherent biases. Future research should explore optimal weighting and normalization strategies, potentially through adaptive mechanisms that dynamically recalibrate weights in training.

### Model drift detection

**Figure 10:**
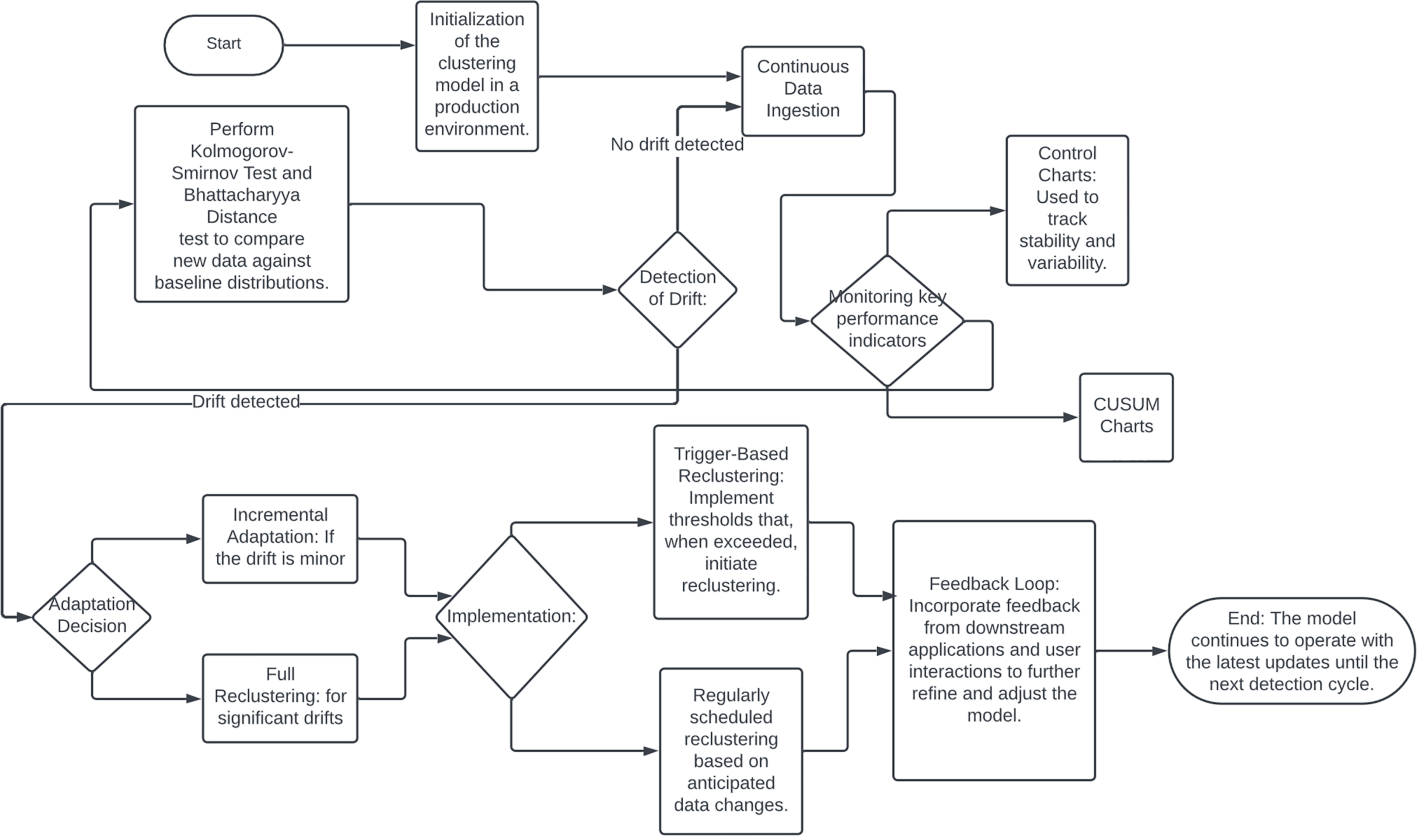
Flowchart of drift detection.

Machine learning models are often trained on historical data under the assumption that the underlying data distribution remains constant over time. However, in real-world scenarios, this assumption frequently does not hold, leading to model drift or concept drift (Quiñonero-Candela et al., 2022). Model drift highlighted on the Fig. 10, occurs when the statistical properties of the target variable change, either gradually or abruptly, causing predictions to become less accurate as the model relies on outdated data (Lu et al., 2018; Ditzler et al., 2015). Model drift poses significant challenges, particularly in clustering algorithms, which are vital for tasks such as customer segmentation, anomaly detection, and data exploration (Quiñonero-Candela et al., 2022). When data distributions shift, the clusters formed from historical data may no longer be valid, leading to unreliable decisions and suboptimal outcomes (Oyelade et al., 2016).

Addressing model drift requires integrating feedback mechanisms into the clustering workflow. Continuous monitoring can detect misalignments between existing clusters and current data distributions, prompting dynamic adjustments or retraining to accommodate new patterns. This adaptation ensures that cluster-based decisions accurately reflect the current data landscape, enhancing segmentation and targeting capabilities. Effective model drift detection and mitigation are also crucial for risk management, especially in regulated industries such as finance and healthcare. Robust drift detection mechanisms help prevent biased algorithmic decisions and ensure compliance with regulatory standards.

Several techniques and methodologies can be employed to detect and adapt to model drift in production environments. Statistical process control methods, such as control charts and CUSUM charts, can monitor clustering model performance and identify outliers or shifts. Data distribution monitoring techniques, including the Kolmogorov-Smirnov test and Bhattacharyya distance, compare incoming data batches against a baseline to detect significant deviations indicating model drift (Ye, Hu & Yu, 2008).

Upon detecting model drift, automated adaptation mechanisms can maintain clustering model accuracy and relevance. Incremental learning algorithms, such as StreamKM++ and CluStream, are designed for streaming data and can adapt to new data in real-time (Aggarwal et al., 2003). Windowing techniques, using a sliding window of recent data, continuously update the clustering model to reflect current trends while discarding outdated information (Khamassi et al., 2015). Trigger-based reclustering, where drift detection metrics initiate a reclustering process or deeper analysis, can also be implemented. Hybrid approaches, including ensemble methods and feedback loops, further enhance clustering model robustness and adaptability (Bifet, Holmes & Pfahringer, 2010). Integrating model drift detection and adaptation into a production environment requires robust infrastructure, including efficient data pipelines, computational resources, and effective visualization and reporting tools.

### Online clustering

Online clustering is used in machine learning where the data is continuously analyzed and clustered in real-time as it arrives, rather than processing the entire dataset in a batch mode (Silva et al., 2013). This approach is particularly useful in situations where data is being generated continuously, such as sensor data streams, financial transactions, or social media feeds (Barbakh & Fyfe, 2008). Online clustering algorithms can handle very large datasets efficiently because they do not require all data to be present in memory at once. They process data points sequentially or in small batches, which significantly reduces memory requirements and computational load compared to traditional batch clustering methods (Bifet, Holmes & Pfahringer, 2010). These algorithms can adapt to changes in the underlying data distribution over time, which is common in dynamic environments (Ditzler et al., 2015). For instance, in consumer behavior analysis or stock market data, patterns can shift due to external influences, and online clustering can adjust to these changes without needing a complete re-run with the entire dataset (Khamassi et al., 2015).

**Figure 11:**
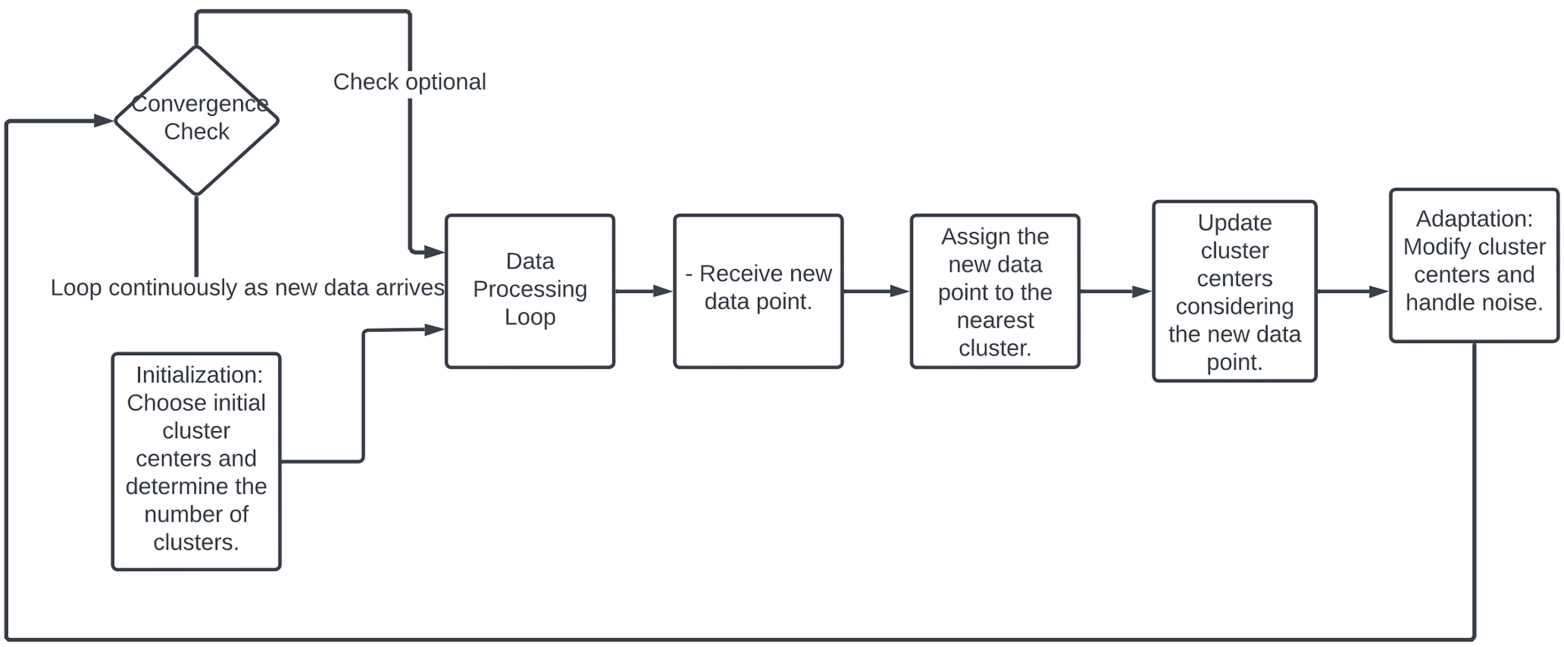
Flowchart of online clustering methodology.

Online clustering provides the capability to analyze and cluster data in real-time. This is crucial for applications that rely on immediate data processing, such as fraud detection systems, where it’s essential to act quickly on the incoming data (Ye, Hu & Yu, 2008). When dealing with high-dimensional data, online clustering can be particularly effective as it allows for incremental feature selection and dimensionality reduction techniques that update as more data becomes available, helping to maintain performance without overwhelming computational resources (Huang, Yoo & Kasiviswanathan, 2015). Online clustering, highlighted on Fig. 11, starts with selecting initial cluster centers randomly or based on a heuristic, with the number of clusters either predetermined or dynamically adjusted (Charikar et al., 1997). As new data points arrive, each is immediately assigned to the nearest cluster based on a distance metric like Euclidean distance. Cluster centers are then updated to incorporate the new data, often by recalculating the mean of all points in each cluster (Huang, Yoo & Kasiviswanathan, 2015). The clustering process continuously adapts by adjusting cluster centers in response to new patterns or changes in the data distribution and by integrating mechanisms to handle noise and outliers. Although some online clustering algorithms may check for convergence, the process typically runs indefinitely, continually adapting as more data flows in Liberty, Sriharsha & Sviridenko (2016).

### Mini-batch clustering

In production environments with large-scale datasets, traditional clustering algorithms face significant bottlenecks due to memory constraints and computational demands. The K-means algorithm, a prevalent method for grouping data into clusters, exemplifies these challenges. Its iterative process involves assigning data points to the nearest cluster centroid, recalculating centroids as the mean of all assigned points, and repeating until convergence. Handling extensive datasets in memory for each iteration and updating centroids by calculating distances for all data points becomes impractical due to the resource-intensive operations required (Lloyd, 1982). To address these limitations, mini-batch clustering, particularly the mini-batch K-means method, has emerged as a pivotal solution. This approach mitigates the constraints of traditional algorithms by processing data in smaller subsets or mini-batches.

**Figure 12:**
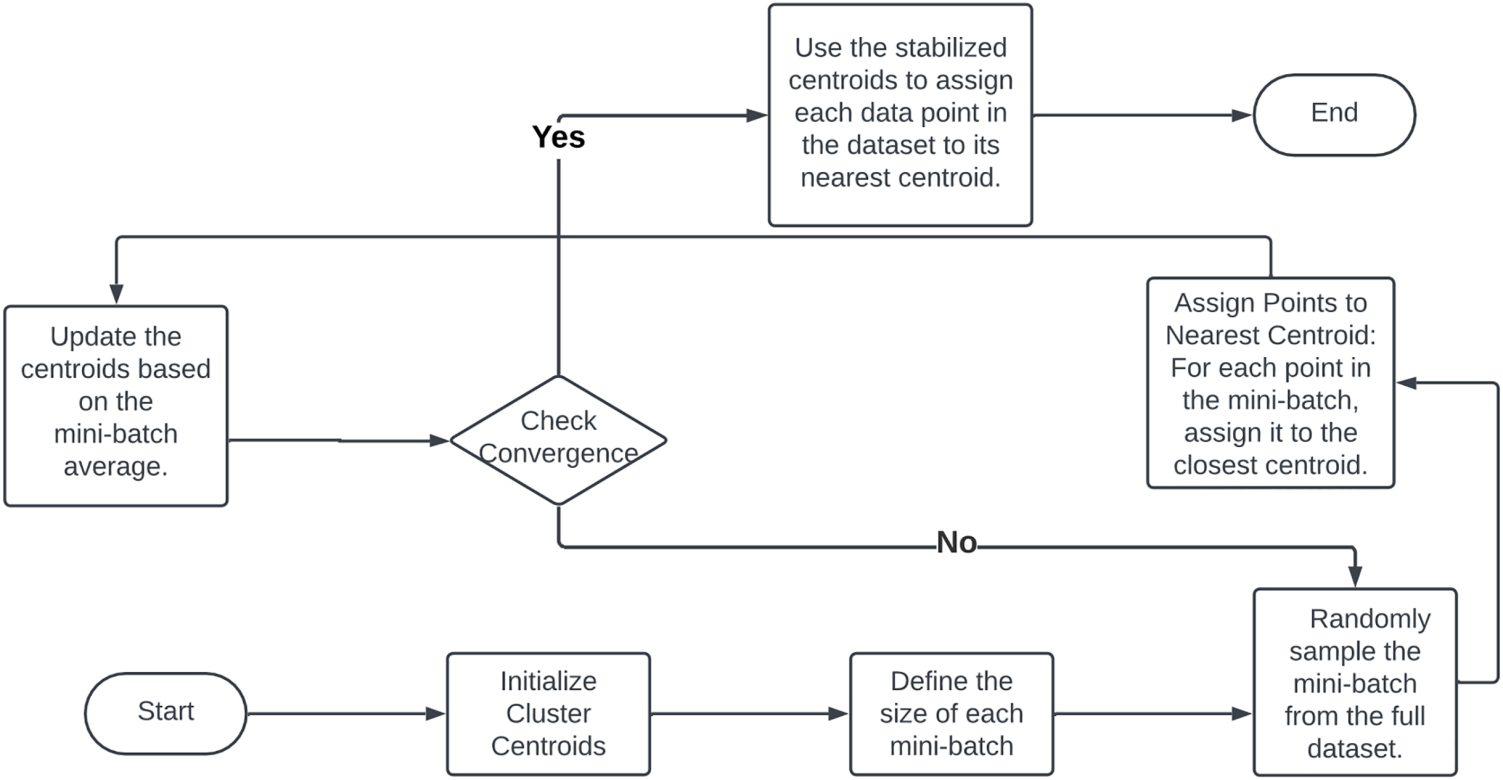
Flowchart of minibatch clustering methodology.

The mini-batch clustering process, highlighted in a flowchart posted on Fig. 12 begins by partitioning the dataset into numerous small batches. Each iteration randomly selects a mini-batch from the dataset, using only the data points within that batch for cluster assignment and centroid updates (Sculley, 2010). This process iterates with new mini-batches until minimal centroid changes are observed, indicating convergence. Mini-batch K-means significantly reduces memory load by processing only a fraction of the data at a time, making it feasible to handle even the largest datasets (Bahmani et al., 2012). The use of smaller data batches enables faster iterations over the entire dataset, leading to quicker convergence compared to the traditional K-means algorithm (Bottou, 1998). The stochastic nature of mini-batches introduces randomness that aids in escaping local optima, potentially resulting in more optimal clustering solutions (Arthur, 2007). Mini-batches mitigate the impact of outliers and noise, as each batch update averages out such anomalies, leading to more stable and robust clusters. The reduced computational complexity of each step, due to the smaller batch sizes, conserves time and processing resources, thereby enhancing the algorithm’s overall efficiency. The benefits of mini-batch clustering extend beyond the K-means algorithm and can be adapted to other clustering techniques (Xu & Tian, 2015).

In summary, mini-batch clustering offers a practical solution to the challenges posed by large-scale data in production settings. By processing data in smaller batches, it alleviates memory constraints, accelerates convergence, improves cluster quality, reduces sensitivity to noise, and lowers computational costs. This approach enhances the production readiness of clustering algorithms and enables more efficient and scalable data analysis across diverse applications, empowering organizations to derive valuable insights from their data repositories (Jain, 2010).

### Sample clustering

As the volume and complexity of data increase, traditional clustering algorithms often struggle with the high computational demands and scalability issues posed by large datasets. Sample-based clustering algorithms provide a robust solution, employing techniques like subsampling to enhance the efficiency and scalability of clustering. These algorithms optimize the data analysis process by reducing the amount of data processed, thus facilitating more effective data management and analysis in large data environments.

**Figure 13:**
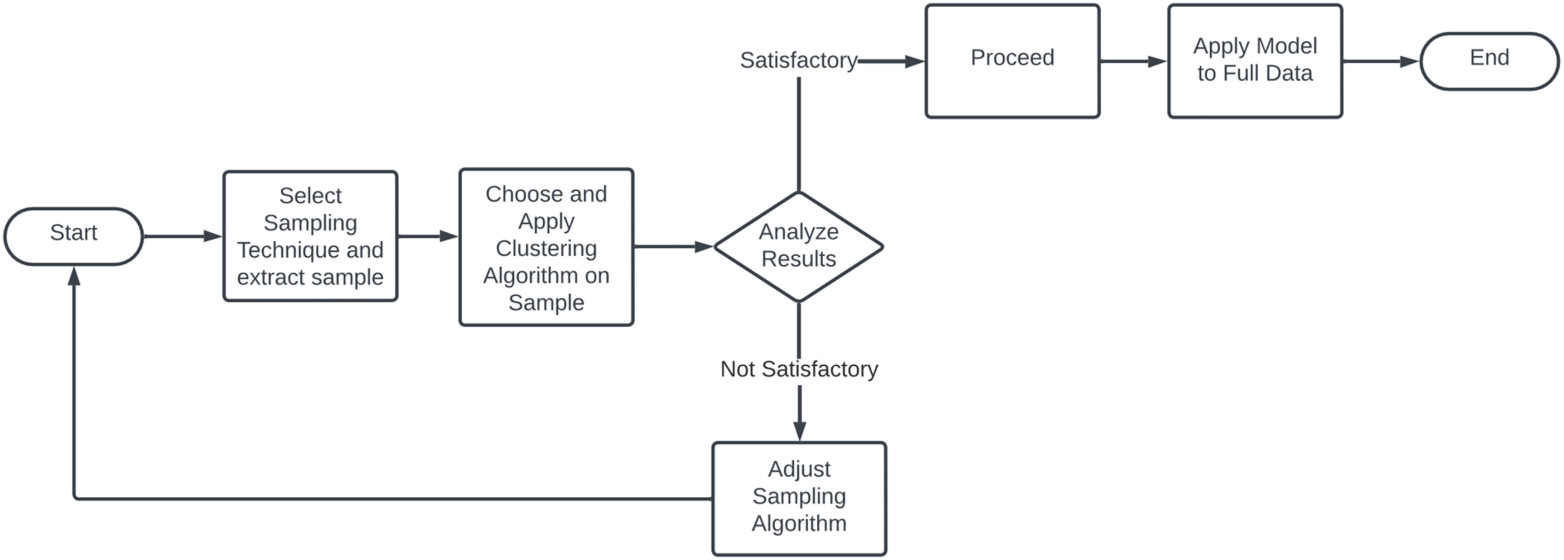
Flowchart of sample clustering methodology.

Sample-based clustering shown here flowchart on Fig. 13 is the strategic reduction of data processed in each iteration. By carefully selecting a representative subset of the overall dataset, these algorithms significantly reduce the computational load while maintaining the accuracy of clustering results. The process typically starts with the application of traditional clustering methods such as K-means or DBSCAN to a sampled subset to identify preliminary cluster centers. These centers are then expanded to the full dataset by assigning each unsampled point to the nearest cluster center. An optional refinement phase may follow, where cluster centers are iteratively adjusted by incorporating more data points, enhancing the clustering accuracy. A major benefit of this approach is its ability to reduce sensitivity to initial conditions, a common issue in algorithms like K-means. By using a representative subset for initial centroid selection, sample clustering minimizes the impact of outliers and anomalies, thereby improving the clustering process (Wang, Gittens & Mahoney, 2019; Chitta et al., 2011). The main sampling strategies are:

- **Random sampling** involves selecting a random subset of the dataset, offering a simple yet effective approach. This method enhances the randomness and coverage of the dataset, reducing bias in the clustering outcomes.
- **Stratified sampling** divides the dataset into strata based on key characteristics, ensuring proportional representation of each stratum in the sample. This method enhances the representativeness of the sample, particularly in heterogeneous datasets.
- **Reservoir sampling** is well-suited for streaming data, employing a fixed-size reservoir to ensure equal probability of inclusion for every data point, despite the continuous influx of data (Efraimidis & Spirakis, 2006). It’s particularly useful in environments where data is dynamically changing.
- **Adaptive re-sampling in dynamic environments** In environments where noise and data dynamics are prevalent, sample clustering offers a distinct advantage. Allowing periodic re-sampling and re-clustering, model can adaptively refine the cluster centers to better reflect the current data state (Charikar et al., 1997). This dynamic sampling approach maintains the clustering model’s relevance and robustness over time, ensuring accurate and reliable results even in the face of evolving data.

Sampling techniques when coupled with standard clustering processes not only enhance operational efficiency but also improves the accuracy and robustness of the results. By strategically reducing the operational data volume through sampling, the computational demands associated with distance calculations and cluster updates are substantially diminished. This approach enhances the scalability of the algorithms and reduces execution time, which is particularly crucial when dealing with high-dimensional data (Liang et al., 2018). For *e.g*., Hierarchical Sample Clustering recursively divides the dataset into smaller subsets, and clustering is performed at each level.

### Caching strategies in production environments

In production environments, where efficiency and speed are paramount, caching strategies are essential for optimizing computational resources for clustering algorithms. Caching, by storing intermediate results of computationally intensive operations, can drastically reduce the time complexity of repeated calculations, thereby enhancing the overall performance of clustering processes.

- **Distance computation caching:** Distance computations in clustering algorithms like K-means and HAC can be computationally expensive, especially in high-dimensional spaces (Jain, Murty & Flynn, 1999; Xu & Tian, 2015). Caching these calculations in a distance matrix or hash table reduces this burden (Fahim et al., 2006). The algorithm checks the cache for previously computed distances, avoiding redundant calculations (Fahim et al., 2006). This approach speeds up convergence and enhances efficiency and scalability for large datasets (Zhou et al., 2008).
- **Centroid calculation caching** significantly improves the efficiency of centroid-based clustering algorithms like K-means (Jain, 2010). By storing the mean values of clusters and using a hash-based caching mechanism, the algorithm avoids redundant calculations for unchanged clusters, only recalculating means for modified clusters (Drake & Hamerly, 2012). This streamlines the update process, reduces computational burden, and accelerates convergence.
- **Memoization in hierarchical clustering (HAC)** characterized by the iterative merging of clusters based on distance metrics, benefits significantly from memoization. This caching technique involves storing previously computed distances between clusters in a memoization table. Consequently, the algorithm can avoid recalculating distances between the same cluster pairs in subsequent iterations, optimizing the HAC process. This strategy is particularly effective in reducing the overall time complexity from potentially quadratic to near-linear, depending on the clustering dynamics.
- **Region query caching for density-based clustering (DBSCAN)** perform region queries to identify dense clusters of points. Caching the results of these queries, particularly in datasets where data points exhibit minimal movement over time, can lead to substantial performance improvements. A spatial index, like an R-tree, can be utilized to efficiently cache and query the spatial data, speeding up the region query process and, by extension, the clustering operation.
- **Distributed caching for Scalability:** Distributed caching systems like Redis enhance clustering scalability by sharing cached data across multiple servers. This approach supports scalable implementations of algorithms like BIRCH, maintaining high data retrieval speeds and efficiently updating clustering feature summaries in real-time applications (Zhang, Ramakrishnan & Livny, 1996).
- **Dynamic cache management** involves automatically adjusting cache sizes and deletion considering current workload and system performance, ensures efficient memory utilization. For clustering this approach prioritizes the caching of critical operations such as distance calculations or centroid updates that significantly impact performance while evicting stale or less frequently accessed data.
- **Selective caching for critical computations:** In memory-constrained environments, selectively caching only the most computationally intensive operations optimizes processing speed without overwhelming system memory. This strategy ensures caching benefits are maximized for operations that significantly contribute to the computational overhead of clustering algorithms.

### Internal validation metrics for optimal performance of clustering models in production

Evaluating the performance of clustering algorithms, which are unsupervised learning models, presents unique challenges. Rather than immediate numerical quantification, a comprehensive data-driven approach is necessary, particularly in dynamic data environments where datasets continuously evolve. These metrics assess the data structure as represented by the model, ensuring that the clusters formed are meaningful and relevant. Below, we detail this framework.

- **Silhouette coefficient:** measures the similarity of an object within its own cluster compared to other clusters. It provides a succinct graphical representation of how well each object has been classified. The coefficient ranges from −1 to 1, where a higher value closer to 1 indicates well-defined and well-matched clusters, suggesting that each data point is more similar to its own cluster than to others and poorly matched to neighboring clusters (Rousseeuw, 1987). Mathematically, this is:
(19) $${\mathrm{Silhouette}}\;{\mathrm{Coefficient = }}{{{{b}}({\mathrm{i}}){{ - a}}({\mathrm{i}})} \over {\max \{ {{a}}({\mathrm{i}}),{{b}}({\mathrm{i}})\} }}.$$
  where $a(i)$ is the mean intra-cluster distance (the average distance from $i$ to all other points in its cluster), and $b(i)$ is the mean nearest-cluster distance (the average distance from $i$ to all points in the nearest cluster).
- **Calinski-Harabasz Index:** also known as the Variance Ratio Criterion, evaluates the compactness and separation of the clusters by comparing the sum of between-cluster dispersion to within-cluster dispersion (Caliński & Harabasz, 1974). High values typically indicate that the clusters are dense and well-separated. Mathematically, this is expressed as:
(20) $${\mathrm{Calinski \!-\! Harabasz}}\;{\mathrm{Index = \,}}{{{B}} \over {{W}}} \times {{{{N - K}}} \over {{{K - 1}}}}$$
  where B represents the trace of the between-cluster dispersion matrix and ${\mathrm{trace}}(W)$ represents the trace of the within-cluster dispersion matrix. *N* is the total number of data points, and $k$ is the number of clusters.
- **Davies-Bouldin Index**: is particularly effective in identifying sets of clusters that are well-separated. The Davies-Bouldin Index is defined as the average ‘similarity’ between each cluster and the most similar one, where similarity is the ratio of within-cluster distances to between-cluster distances. Lower values of the Davies-Bouldin Index indicate a clustering configuration with better separation between the clusters (Davies & Bouldin, 1979). Mathematically, this is expressed as:
(21) $${\mathrm{Davies \!-\! Bouldin}}\;{\mathrm{Index = }}{{\mathrm{1}} \over {{K}}}\sum\limits_{{{i = 1}}}^{{K}} {{\mathop{\max }_{{{j}} \ne {{i}}}}} \left( {{{{\sigma _{{i}}}{{ + }}{\sigma _{{j}}}} \over {{{d}}({{{c}}_{{i}}},{{{c}}_{{j}}})}}} \right)$$
  where ${c_i}$ and ${c_j}$ are the centroids of clusters $i$ and $j$ respectively. ${\sigma _i}$ and ${\sigma _j}$ are the average distances of points in clusters $i$ and $j$ respectively to their respective centroids. $d({c_i},{c_j})$ is the distance.
- **Dunn Index** is designed to identify compact and well-separated clusters. It is defined as the ratio of the minimum inter-cluster distance to the maximum intra-cluster distance (Ncir, Hamza & Bouaguel, 2021). The minimum inter-cluster distance is the shortest distance between the centroids of any two clusters, and the maximum intra-cluster distance is the diameter of the largest cluster. A higher Dunn Index indicates well-separated and compact clusters. This is calculated by the ratio of the minimum inter-cluster distance to the maximum intra-cluster distance. Mathematically Dunn index is expressed as:
(22) $$D = {{{{\min }_{1 \le i{\mathrm{ \lt}}j \le k}}d({c_i},{c_j})} \over {{{\max }_{1 \le l \le k}}\delta ({\omega _l})}}.$$
  where $d({c_i},{c_j})$ is the distance between centroids of clusters $i$ and $j$, and $\delta ({\omega _l})$ is the diameter of cluster $l$, defined as the maximum distance between any two points within the cluster.
- **Gap statistics** estimates the optimal number of clusters by comparing the within-cluster dispersion for different numbers of clusters with their expected values under a null reference distribution. The within-cluster dispersion for the observed data is compared to the within-cluster dispersion for multiple reference datasets generated from a uniform distribution. The optimal number of clusters is the $k$ that maximizes the Gap Statistic, indicating significant deviation from randomness (Tibshirani, Walther & Hastie, 2001). For a given number of clusters $k$, the gap statistic is defined as:
(23) $${\mathrm{Gap}}(k) = {1 \over B}\sum\limits_{b = 1}^B {\log } (W_k^b) - \log ({W_k})$$
  where ${W_k}$ is the within-cluster dispersion for the observed data, and $W_k^b$ is the within-cluster dispersion for the $b$-th reference dataset generated from a uniform distribution.
- **Density based clustering validation (DBCV)** is tailored for evaluating density-based clustering algorithms, particularly those capable of identifying clusters with arbitrary shapes, including concave clusters. DBCV measures the ratio of the density within clusters to the density between clusters. The density of a point within its cluster is defined based on the local reachability density (LRD), which is calculated as the inverse of the average distance from the point to its k-nearest neighbors, with a parameter to prevent division by zero. The DBCV is then calculated as the average ratio of the difference between the LRD within clusters and the LRD between clusters to the maximum of these two densities (Moulavi et al., 2014). A higher DBCV value indicates better clustering performance, especially for complex cluster shapes. Mathematically, this is expressed as:
(24) $$LRD(p) = {1 \over {{1 \over {|{N_k}(p)|}}\sum\nolimits_{o \in {N_k}(p)} {{\mathrm{reach - dis}}{{\mathrm{t}}_k}} (p,o)}}$$
  where ${N_k}(p)$ is the set of $k$-nearest neighbors of point $p$, and ${\mathrm{reach - dis}}{{\mathrm{t}}_k}(p,o)$ is the reachability distance between points $p$ and $o$. The DBCV is computed as:
(25) $$DBCV = {{\sum\nolimits_{i = 1}^k {\sum\nolimits_{p \in {C_i}} {{{LRD(p) - LR{D_{{\mathrm{out}}}}(p)} \over {\max \{ LRD(p),LR{D_{{\mathrm{out}}}}(p)\} }}} } } \over {\sum\nolimits_{i = 1}^k | {C_i}|}}$$
  where $LR{D_{{\mathrm{out}}}}(p)$ represents the local reachability density for the closest point outside the cluster ${C_i}$. Higher DBCV values indicate better performance, especially for complex cluster shapes.

These metrics quantify cluster coherence and separation, objectively assessing clustering performance without labeled data. They help gauge internal validity, facilitating model tuning to adapt to evolving data and business needs. Considering computational complexity and scalability is vital for production deployment. Efficient, parallelizable implementations are crucial as dataset sizes grow and real-time processing becomes more prevalent. In summary, the combination of the silhouette coefficient, Calinski-Harabasz Index, Davies-Bouldin Index, Dunn Index, Gap Statistic, and DBCV provides a comprehensive framework for evaluating clustering algorithms. Each metric offers unique insights into different aspects of cluster quality, from cohesion and separation to density and shape. Utilizing these metrics ensures a thorough assessment of clustering quality and can guide the selection and optimization of clustering algorithms for various applications.

### Continuous monitoring and integration with business operations

In the ever-evolving landscape of data-driven industries, continuous monitoring and strategic integration of clustering models with business operations are paramount. This ensures not only the statistical robustness of the models but also their alignment with organizational goals, delivering measurable business value.

- **Stability metrics:** To gauge the reliability of clustering models amidst fluctuating data patterns, stability metrics assess the consistency of cluster assignments over time or across data snapshots. For *e.g*., the Adjusted Rand Index (ARI) offers a normalized measure to compare the similarity between two cluster assignments, highlighting shifts in cluster stability due to evolving data (Larsen & Aone, 1999). Where $a$ is the number of pairs in the same cluster for both observed and predicted clusterings, $b$ is the number of pairs in different clusters for both, $c$ is the number of pairs in the same cluster for observed but different for predicted, and $d$ is the number of pairs in different clusters for observed but the same for predicted.
(26) $$RI = {{a + b} \over {a + b + c + d}}.$$
- **Anomaly detection** Incorporating anomaly detection techniques, such as Isolation Forests, enables early identification of outliers indicating shifts in data distribution. This proactive measure can signal the need for model recalibration to adapt to new data behaviors, ensuring the ongoing relevance of clustering models (Liu, Ting & Zhou, 2008).
- **A/B Testing and Multi-Arm Bandits** Conducting controlled A/B testing, and multi-armed bandit algorithms, allows for real-time comparison of clustering strategies. Unlike traditional A/B testing, which compares a static set of models, multi-armed bandit approaches continuously adjust the allocation of resources to models based on their performance, optimizing for the best outcome and significantly reducing the time to identify the most effective clustering model (Scott, 2015).
- **Integration with KPIs** Directly correlating clustering model performance with key performance indicators (KPIs) such as customer retention rates or average revenue per user (ARPU) ensures that the models contribute to strategic business objectives. This integration necessitates the development of utility scores, which quantify the impact of clustering decisions on these KPIs, offering a tangible measure of the models business value.

Integrating these strategies into the clustering life-cycle ensures their utility in production. By prioritizing continuous model validation through stability metrics, anomaly detection, and A/B testing, and aligning outcomes with business KPIs, organizations can maintain the relevance and strategic value of their clustering models. Continuous monitoring and integration pave the way for adaptive, self-tuning models that dynamically adjust parameters based on real-time feedback.

## Conclusion and future work

Although clustering algorithms have evolved considerably, several challenges persist that must be addressed to enhance their utility in practical scenarios. Key among these challenges is the complexity associated with high-dimensional, dynamic, and noisy datasets. Future efforts should focus on refining dimensionality reduction techniques and robust statistical methods to better cope with these complexities. Moreover, as data volumes expand, the scalability and computational efficiency of clustering algorithms will become increasingly critical. Enhancements in distributed computing and the use of GPU-accelerated algorithms are vital for supporting real-time processing and analysis of large-scale datasets.

**Figure 14:**
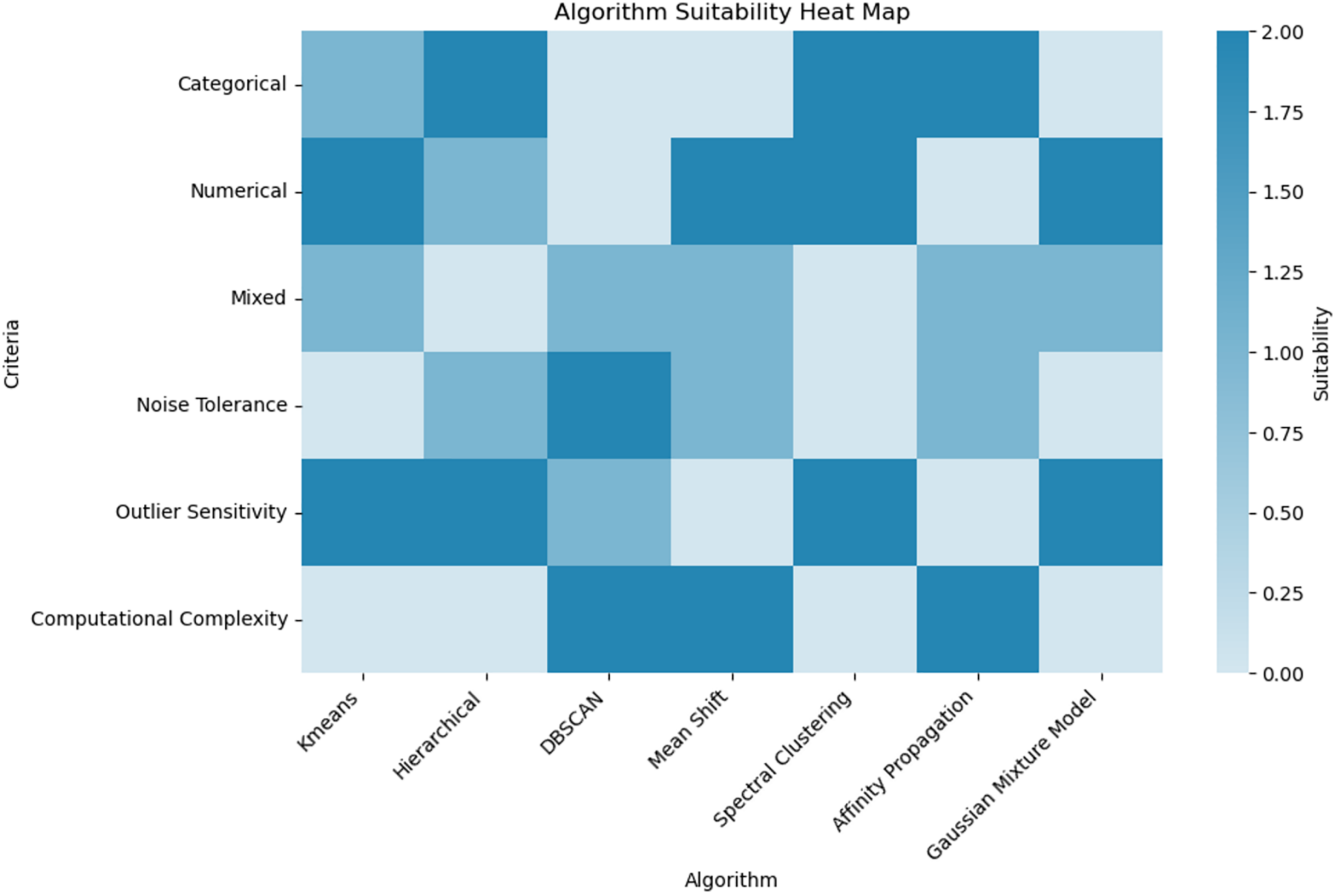
Algorithm suitability heat map for clustering techniques. This heat map provides a comparative analysis of various clustering algorithms against specific criteria relevant to data type and algorithm performance. Each cell is color-coded to indicate the suitability of an algorithm for a given criterion, ranging from light to dark blue. The criteria evaluated include the handling of categorical, numerical, and mixed data types, noise tolerance, outlier sensitivity, and computational complexity. This visual representation aids in selecting the most appropriate clustering algorithm based on specific requirements and data characteristics.

The interpretability and explainability of clustering outcomes, especially in intricate data landscapes, continue to demand attention. For instance, simplified visualizations such as the heatmap in Fig. 14 can help demystify the outcomes of complex clustering algorithms. Developing frameworks that provide transparent insights into these algorithms will be crucial for fostering trust, particularly in sensitive sectors like healthcare and finance. Additionally, the ability to adapt to streaming and dynamically changing data will be essential. This calls for further research into incremental and adaptive clustering techniques that can update models in response to new data inputs.

The findings of this survey have significant practical implications across various fields. For example, in bioinformatics, advanced clustering algorithms can improve the accuracy of gene expression analysis and disease classification. In image segmentation, these techniques can enhance the precision of object detection and recognition. Additionally, in customer segmentation, improved clustering methods can lead to more targeted marketing strategies and better customer insights. By addressing key challenges and proposing advanced solutions, this survey provides a framework for developing more efficient and adaptable clustering algorithms that can handle the complexities of real-world data, ultimately leading to more effective and actionable insights in various applications.

Looking forward, the integration of clustering algorithms with emerging technologies such as deep learning and quantum computing presents a promising avenue for overcoming existing limitations. Deep learning can enhance the feature extraction capabilities and scalability of clustering algorithms, while quantum computing offers potential breakthroughs in processing speed and efficiency. Another critical area for future research is the development of adaptive algorithms that can dynamically adjust to evolving data streams in real-time applications such as Internet of Things (IoT) and social media analytics.

Furthermore, ensuring the privacy and security of data in clustering processes, especially in sensitive fields like healthcare and finance, will become increasingly important. Techniques that provide robustness against adversarial attacks and guarantee data integrity need to be developed. Additionally, the interpretability of clustering results, crucial for decision-making in domains like bioinformatics and marketing, needs enhancement. Developing methods that offer clearer insights into cluster formations and their characteristics will aid in bridging the gap between technical outputs and actionable insights.

In conclusion, while significant progress has been made in the clustering domain, substantial challenges remain. Addressing these challenges through innovative research and interdisciplinary collaboration will be key to unlocking the full potential of clustering algorithms across various fields. Future research should also prioritize user-friendly and scalable implementations to facilitate broader adoption and application.

## Additional Information and Declarations

### Data Availability

The following information was supplied regarding data availability: This is a literature review.
